# Her6 and Prox1a are novel regulators of photoreceptor regeneration in the zebrafish retina

**DOI:** 10.1371/journal.pgen.1011010

**Published:** 2023-11-06

**Authors:** Kellie Veen, Aaron Krylov, Shuguang Yu, Jie He, Patrick Boyd, David R. Hyde, Theo Mantamadiotis, Louise Y. Cheng, Patricia R. Jusuf

**Affiliations:** 1 Peter MacCallum Cancer Centre, Melbourne, Victoria, Australia; 2 Sir Peter MacCallum Department of Oncology, The University of Melbourne, Melbourne, Victoria, Australia; 3 School of BioSciences, The University of Melbourne, Melbourne, Victoria, Australia; 4 Institute of Neuroscience, State Key Laboratory of Neuroscience, CAS Centre for Excellence in Brain Science and Intelligence Technology, Chinese Academy of Sciences, Shanghai, China; 5 Department of Biological Sciences, Center for Zebrafish Research, and Center for Stem Cells and Regenerative Medicine, University of Notre Dame, Notre Dame, Indiana, United States of America; 6 Department of Microbiology and Immunology, The University of Melbourne, Melbourne, Victoria, Australia; 7 Department of Anatomy and Physiology, The University of Melbourne, Melbourne, Victoria, Australia; HudsonAlpha Institute for Biotechnology, UNITED STATES

## Abstract

Damage to light-sensing photoreceptors (PRs) occurs in highly prevalent retinal diseases. As humans cannot regenerate new PRs, these diseases often lead to irreversible blindness. Intriguingly, animals, such as the zebrafish, can regenerate PRs efficiently and restore functional vision. Upon injury, mature Müller glia (MG) undergo reprogramming to adopt a stem cell-like state. This process is similar to cellular dedifferentiation, and results in the generation of progenitor cells, which, in turn, proliferate and differentiate to replace lost retinal neurons. In this study, we tested whether factors involved in dedifferentiation of *Drosophila* CNS are implicated in the regenerative response in the zebrafish retina. We found that *hairy-related 6* (*her6*) negatively regulates of PR production by regulating the rate of cell divisions in the MG-derived progenitors. *prospero homeobox 1a* (*prox1a*) is expressed in differentiated PRs and may promote PR differentiation through phase separation. Interestingly, upon Her6 downregulation, Prox1a is precociously upregulated in the PRs, to promote PR differentiation; conversely, loss of Prox1a also induces a downregulation of Her6. Together, we identified two novel candidates of PR regeneration that cross regulate each other; these may be exploited to promote human retinal regeneration and vision recovery.

## Author summary

Nerve cells in the central nervous system of humans and other mammals do not regenerate after damage (e.g. brain injury or genetic degenerative disorders). Damage to the visual neurons for instance often results in complete and irreversible blindness. Here, we used the regenerative zebrafish model to investigate novel genes regulating this response. We identify two genes that are expressed at separate times following injury, specifically in the regenerative stem cells of the visual system. By disrupting the expression of these, we showed novel important roles in controlling stem cell divisions (required to make more cells) and in nerve cell maturation (required to regenerate the fully functioning nerve cells). Both are ancient genes present in many evolutionary distant animals and were identified in our study by their ability to affect stem cells in the fruit fly brain.

## Introduction

Vision is initiated in the eye by specialised light-detecting cells termed photoreceptors (PRs). PRs are sensory neurons that convert light into electrical signals which are transmitted to subsequent neural circuits and interpreted by the brain. PRs can be classified as either rods, which detect low levels of light, allowing for low-level scotopic vision such as night vision; or cones, which detect specific wavelengths of light at greater intensities, conferring photopic vision such as colour vision. Rods and cones are highly specialised and consist of complex and precise structures that each carry out specific functions. Both PR types are comprised of a light-sensitive outer segment, a connecting cilium, a cell body, and a synaptic terminal. Due to the complex nature of PR development, as well as their high metabolic and functional demands, PRs are vulnerable to mutations and prone to visual diseases. In the disease retinitis pigmentosa, PRs within the retina degenerate over time [1]. As human retinal cells cannot regenerate, this vision loss is irreversible. Unlike humans, zebrafish retinae can regenerate after injury. Therefore, it is key to understand how zebrafish regeneration occurs, such that potential therapies to regenerate lost human PRs can be developed.

Zebrafish retinal regeneration is achieved by the reprogramming of mature Müller glia (MG) into a stem-like state, and subsequent production of progenitor cells that differentiate into mature neurons such as PRs [2–7]. Dedifferentiation, the process in which mature cell types are reprogrammed and adopt stem-like properties [8–10], is a crucial mechanism in regeneration, as it enables the acquisition of a stem-like fate in mature cells. Therefore, factors implicated in dedifferentiation may be involved in regeneration.

In this study, we found two transcription factors that regulate dedifferentiation within the *Drosophila* brain are involved in regeneration of the zebrafish retina. Hairy-related 6 (Her6), the zebrafish ortholog of *Drosophila* Deadpan (Dpn), and Prospero homeobox 1a (Prox1a), the zebrafish ortholog of Prospero (Pros) mediate the regenerative response following a metronidazole inducible transgenic photoreceptor (red cones) ablation injury model. The regenerative response involves MG acquisition of stem cell fate, proliferation, migration, differentiation, and integration into the functional circuit [11]. We found that Her6 may negatively inhibit progenitor cell divisions, whereas Prox1a is required to promote PR differentiation, and this may occur via phase separation. Her6 is downregulated in the MG-derived progenitors and its knockdown was sufficient to increase the number of PRs after injury. Prox1a was expressed in the differentiating PRs and knockdown of Prox1a resulted in reduced PR differentiation. Additionally, Prox1a expression and PR number was affected by the manipulation of Liquid-Liquid Phase Separation (LLPS). Taking these observations together, we propose novel roles for Her6 and Prox1a in PR regeneration.

## Materials and methods

### Ethics statement

Zebrafish were maintained within the *Danio rerio* University of Melbourne (DrUM) fish facility at the University of Melbourne (ethics approval ID10400, 22235), in accordance with local guidelines. All experimental procedures were approved by the Faculty of Science Animal Ethics Committee at the University of Melbourne (ethics approval ID10232).

### Fly husbandry and strains

*Drosophila melanogaster* stocks were maintained on standard medium at room temperature (22°C). Overexpression and knockdown experiments were set up at 25°C, and after 48 hours, the progeny was heat shocked for 15 minutes at 38°C and moved to 29°C.

The fly strains used were: *hsFLP*, *act>CD2> Gal4; UAS-Dcr2*, *UAS-GFP/ TM6b*, *prospero RNAi/ CyoYFP; UAS GFP/ MKRS*, *UAS-deadpan* (A. Baonza), *UAS-elav RNAi* (#37915, VCRC), *UAS-mCherry RNAi* (#35785, Bloomington), *UAS-SoxNeuro RNAi* (#25996, Bloomington).

### Immunostaining fly samples

At five days after larval hatching, larvae were dissected in phosphate buffered saline (PBS), fixed in 4% formaldehyde for 20 minutes, and washed in PBS containing 0.5% Triton X 100 (PBST). Tissues were incubated in a primary antibody solution overnight at 4°C, followed by an overnight incubation of secondary at 4°C. Samples were mounted in 80% glycerol in PBS for image acquisition. Primary antibodies used were mouse anti-Mira (1:50; gift of Alex Gould) and chick anti-GFP (1:2000; Abcam). Secondary donkey antibodies conjugated to Alexa 555 and goat antibodies conjugated to Alexa 488 (Molecular Probes) were used at 1:500.

### Fish husbandry and set up

Adult zebrafish were kept at 28.5°C on 12/12h hour light/dark cycles. Zebrafish lines used were Tg(*lws2*:*nfsb-mCherry*^*uom3Tg*^) [12,13], Tg(*gfap*:*eGFP*)^mi2001^ [14]. For breeding, 2–4 males and females were set up in breeding tanks overnight, separated by a clear barrier, which was removed the next morning. Fertilized eggs were collected in E3 (5mM NaCl; 0.17mM KCl; 0.33mM CaCl_2_; 0.33mM 178 MgSO_4_) at a maximum density of 50 eggs/40mL petri dish.

### Morpholino injection and electroporation

Protocol as per Thummel et al., 2011 [15]. Morpholino (MO) oligos were custom-designed with a 3’-Lissamine end modification and ordered through GeneTools (Philomath, OR). MOs were injected into adult (11–13 months) zebrafish retinas, followed by electroporation and recovery. Treatment fish were then immediately subjected to injury, controls were kept in fish water.

### MO sequences

Lissaminated Standard Control MO (CCTCCTACCTCAGTTACAATTTATA; 25)

*her6* MO (TATCGGCAGGCATCTTCTCTGGGAA; 25)

*prox1a* MO (ATGTGCTGTCATGGTCAGGCATCAC; 25)

### Zebrafish injury model

Tg(*lws2*:*nfsb-mCherry*) was used as a metronidazole (MTZ) inducible photoreceptor ablation line. Long wavelength sensitive (*lws2*) promoter drove expression of the oxygen-insensitive NAD(P)H Nitroreductase (nfsB), encoding the Nitroreductase (NTR) enzyme fused to the mCherry fluorescent reporter protein. NTR converted MTZ into a cytotoxin, causing DNA mutations and resulting in precise ablation of the targeted neurons [16], which could be visualised by loss of the mCherry tag. In order to induce ablation, fish were swum in 8mM (larvae) or 10mM (adult) MTZ in fish water for 24 hours at 28.5C.

### Zebrafish drug treatments

Larvae were swum in 1.5% or 5% 1,6-Hexanediol (Sigma, #240117) or 2,5-Hexanediol (Sigma, #H11904) in fish water for two, ten, or thirty minutes at 3-6dpf, before returning to fish water.

### Husbandry of adult zebrafish in laboratory during experiments

Starting with the electroporation day, adult fish were not fed. After the procedure, fish were kept off constant water flow in 28.5°C incubators with 12h/12h light/dark cycle. They were monitored twice daily (including pH and ammonia test) and the water was changed daily for 1–5 days preceding their humane death.

### Immunostaining fish samples

Fish were humanely killed between 4–7 days post ablation (dpa) by overdose with 1000 ppm AQUIS (Primo Aquaculture, #106036). For adults, eyes were dissected and fixed in 4% paraformaldehyde (PFA) in phosphate-buffered saline (PBS) at 4°C overnight, while larvae were fixed whole. Samples were washed 3 times for 10 minutes in PBS, immersed in 30% sucrose for 24 hours at 4°C, and immersed in a 50/50 solution of Optimal Cutting Temperature (OCT) compound and 30% sucrose for 24 hours at 4°C. Eyes/whole larvae were embedded in 100% OCT, frozen into moulds using an ethanol/dry ice bath, and stored at -20°C.

Embedded tissue was sectioned using a Leica Cryostat (object temperature -20°C, chamber temperature -16°C). Transverse zebrafish retina sections were cut at 12μm thickness, collected onto Superfrost slides, and left at room temperature to dry before being stored at -20°C.

The sections were defrosted at room temperature before being washed for 10 minutes in PBS. Antigen retrieval was performed for PCNA staining by incubating slides in 2M HCl for 30m at 60°C. Antibodies were diluted in 5% fetal bovine serum (FBS) in PBS. The slides were covered with Parafilm and paper tissue was dampened with PBS, before placing at the bottom of the staining container to provide humidity. After overnight incubation at 4°C, the slides were washed 3 times for 10 minutes in PBS, the secondary antibodies were applied and left at room temperature for 3–4 hours. The sections were then washed 2 times with PBS for 10 minutes each to remove excess secondary antibody. A final 15–20 minute wash containing 100 μ/L 4,6-diamidino-2-phenylindole (DAPI) was performed to stain the nuclei, after which slides were cover-slipped with Mowiol, left to dry overnight at room temperature, and stored at 4°C. Primary antibodies used were mouse anti-Prox1a (1:2000–1:10000, Invitrogen), mouse anti-GS (1:500, Merck MAB302), rabbit anti-PCNA (1:400, Sigma SAB2701819), rabbit anti-Hes1 (1:500, Invitrogen #MA5-32258), mouse anti-zpr1 (1:500, Zebrafish International Resource Centre), rabbit Anti-Hes4 (1: 500, Invitrogen #PA5-84551). Secondary goat antibody conjugated to anti-mouse or anti-rabbit Alexa 488 or 647 (Molecular Probes) were used at 1:500.

### Microscopy and analysis

Slides were imaged using the Olympus FV3000 confocal microscope within the Centre for Advanced Histology and Microscopy (CAHM) at the Peter MacCallum Cancer Centre. A 40x or 60x objective lens (oil immersion) was used to capture images using 405nm, 488nm, 561nm, and 633nm channels. Images were processed with Fiji Is Just ImageJ (Fiji) and Photoshop.

### Statistical analysis

#### Analysis of clones in fly brains

At least eleven animals per genotype were used for all experiments. Volume of clones or regions of interest was estimated from three-dimensional reconstructions of 2 μm spaced confocal Z stacks with Volocity software (Improvision).

Clone volume was calculated by making a Region Of Interest (ROI) around the GFP+ clone (excluding the top section where the superficial NBs are). The rate of dedifferentiation was represented as the volume of Mira+ cells as a percentage of clone.

In all graphs and histograms, error bars represent the standard error of the mean (SEM) and p values are calculated by performing two-tailed, unpaired Student’s t test. The Welch’s correction was applied in case of unequal variances. Each dot on the graphs represents one clone.

#### Analysis of zebrafish retinal sections

At least five animals per genotype were used for all experiments. All analysis was carried out in Fiji using freehand selections and measure tools.

Quantification of the percentage of PCNA+/Prox1a+/zpr1+ cells within the INL or ONL was calculated by manually drawing around the INL/ONL and measuring the area, followed by analysing the mean number of voxels in a given channel within that area. The percentage of PCNA+/Prox1a+/zpr1+/Hes1+ cells within the INL or ONL was therefore represented as the % of positive voxels within a given area. The number of PH3+ cells within the INL/ONL was calculated by manually counting the number of PH3+ cells and normalising it to the area of the INL/ONL.

In all graphs and histograms, error bars represent the standard error of the mean (SEM) and p-values are calculated by performing two-tailed, unpaired Student’s t test. The Welch’s correction was applied in case of unequal variances. Each dot on the graphs represents one retina.

#### Single-cell sample preparation

All single-cell preparation/sequencing/processing was carried out as per Krylov at al., 2023 [17]. Zebrafish line Tg(*her4*.*1*:*dRFP*^*knu2Tg*^*/gfap*:*eGFP*^*mi2001*^*/lws2*:*nfsb-mCherry*^*ion8hTg*^) for red cone ablation, was used in this study [14,18,19]. Zebrafish larvae at 6 days post-fertilisation (dpf) were swum in a 10mM solution of metronidazole for 48 hours, to specifically eliminate the relevant subtype of cone photoreceptor. At 8 dpf, fish were rinsed in fresh system water under standard housing conditions. At 9 dpf (3 days post-injury; dpi), fish were humanely killed. The single-cell suspensions of 9 dpf zebrafish were prepared by following a published protocol [20]. Retinae were dissected and digested in 350 μl papain solution at 37°C for 15 minutes. The papain solution was prepared as follows: 100 μl papain (Worthington, LS003126), 100ul of 1% DNase (Sigma, DN25), and 200 μl of 12mg/ml L-cysteine (Sigma, C6852) were added to a 5 ml DMEM/F12 (Invitrogen, 11330032). During digestion, retinal tissue was mixed by pipetting up and down 4 x 10 times. Following digestion, 1400 μl of washing buffer was added, containing 65 μl of 45% glucose (Invitrogen, 04196545 SB), 50ul of 1M HEPES (Sigma, H4034), and 500 μl FBS (Gibco, 10270106) in 9.385ml of 1x DPBS (Invitrogen, 14190–144). All solutions were filtered through a 0.22 μm filter (Millipore) to sterilise and stored at 4°C prior to use.

#### 10X Chromium single-cell RNA sequencing

To perform single-cell RNA sequencing (scRNA-seq), FACS-isolated cells were loaded onto the Chromium Single Cell Chip (10x Genomics, USA) according to the manufacturer’s protocol. The scRNA-seq libraries were generated using the GemCode Single-Cell Instrument and Single Cell 3’ Library and Gel Bread kit v2 and v3 Chip kit (10x Genomics, 120237). Library quantification and quality assessments were performed by Qubit fluorometric assay (Invitrogen) and dsDNA High Sensitivity Assay Kit (AATI, DNF-474-0500). The fragment analyser was performed using the High Sensitivity Large Fragment -50kb Analysis Kit (AATI, DNF-464). The indexed library was tested for quality and sequenced by the Illumina NovaSeq 6000 sequencer with the S2 flow cell using paired-end 150 x 150 base pairs as the sequencing mode. Sequencing depth was 60K reads per cell.

#### Quality filtering and pre-processing

Filtered matrix raw data files were further analysed in R using Seurat. Prior to downstream filtering, 10520 cells were obtained. Post-filtering and exclusion of non-Müller glia-derived cell types outputted achieved 9789. Low quality cells or cells containing doublets were excluded from all datasets; reads between 200 and 4500 genes per cell were included. Cells with a percentage of mitochondrial gene expression of greater than 35% were excluded from the analysis.

Samples were analysed using Seurat::NormalizeData, variable features for downstream analysis were identified using Seurat:: FindVariableFeatures, and scaled using Seurat::ScaleData. An optimal number of principal components (PCs) (generated through Seurat::RunPCA) for dimensional reduction were selected using the function Seurat::ElbowPlot. PCs containing the greatest variance were selected. Out of the 20 PCs originally specified, 15 PCs were selected to guide the unbiased clustering analysis. Clustering was performed using the shared nearest neighbour (SNN), graph-based approach through Seurat::FindNeighbours. Seurat::FindClusters produced a uniform manifold approximation and projection (UMAP) plots containing 12 clusters. Data is provided at NCBI GEO, accession number GSE218107.

## Results

### Candidate genes: *elav*, *dpn*, *pros*, and *SoxN* regulate dedifferentiation in the *Drosophila* medulla

**Fig 1:**
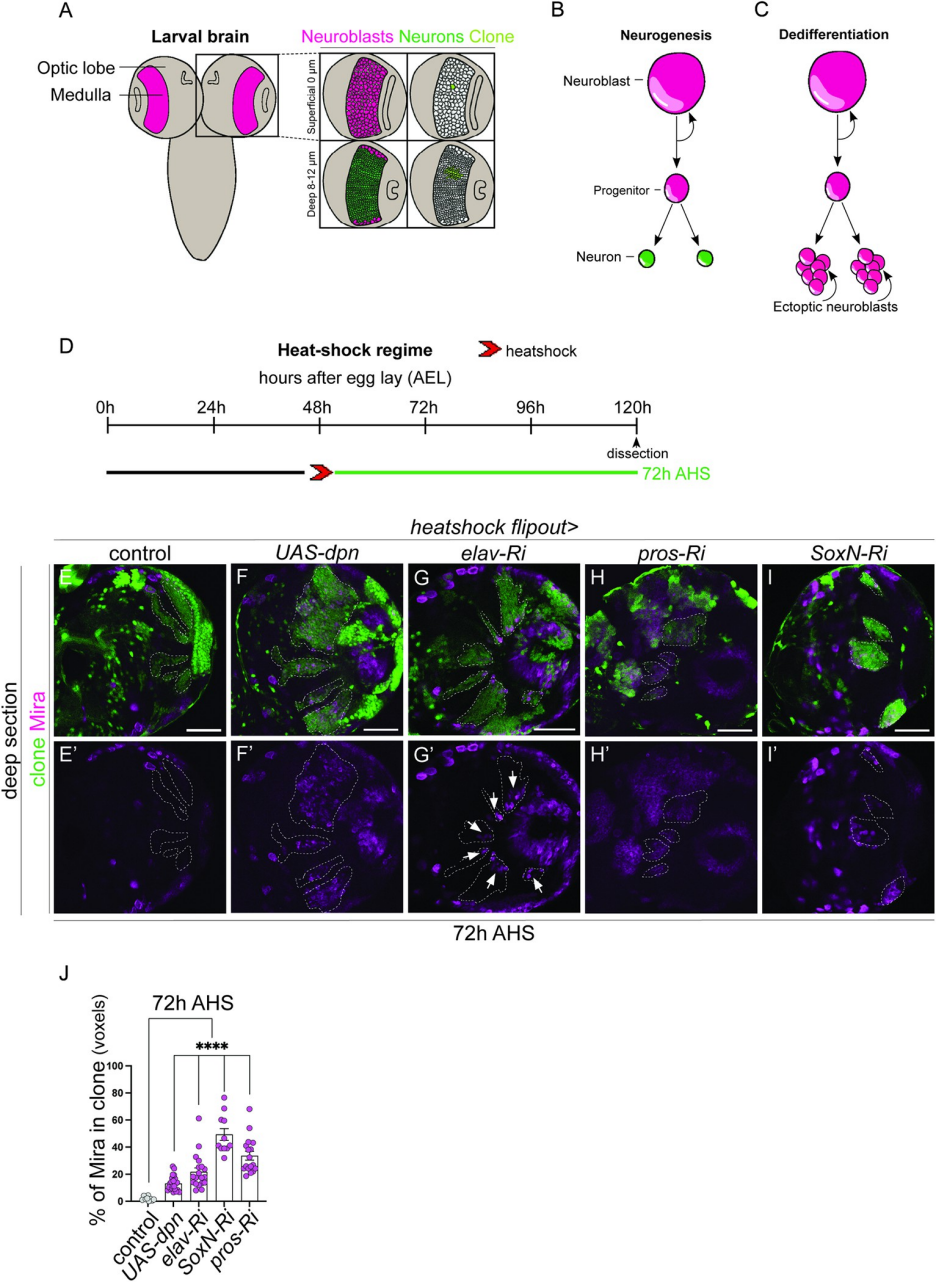
Knockdown of Dpn, Elav, Pros, and SoxN result in dedifferentiation of medulla neurons. (A) Schematic representation of *Drosophila* larval central nervous system (CNS). The medulla (pink) is located with the Optic Lobes (OLs) of the CNS. The superficial surface of the medulla (0μm) contains neuroblasts (NBs, pink), whereas the deep layers (8–12μm) contain mature neurons (green). A single NB in the superficial layer and its neuronal progeny in the deep layers are labelled in light green. (B) During neurogenesis NBs undergo asymmetric divisions to generate a NB and an intermediate progenitor (ganglion mother cell, GMC). The GMC produces 2 neurons. (C) During dedifferentiation, mature neurons dedifferentiate to give rise to ectopic NBs, which continue to divide and create tumours. (D) Experimental design to induce clones with manipulate gene expression. Clones induced via heat shock (red arrows) at 48 hours after egg lay (AEL) are dissected 72 hours after heat shock (AHS). (E-I’) In the deep layers of the medulla, control clones contained no stem cells (E, E’), clonal overexpression of *UAS-dpn (deadpan)*, *elav-Ri (embryonic lethal)*, *pros-Ri (prospero)*, *SoxN-Ri (SoxNeuro)* resulted in stem cell induction (Miranda+, magenta) in the deeper mature neuron layers, indicative of dedifferentiation (J). (J) Quantification of % Mira+ cell volume vs. clone volume: Control (n = 9, 1.874 ±0.4897), *UAS-dpn* (n = 17, 33.62 ±3.243), *elav-Ri* (n = 22, 13.16 ±1.164), *SoxN-Ri* (n = 19, 21.74 ±2.926), *pros-Ri* (n = 11, 49.34 ±4.36). Data are represented as mean ± SEM. ****p < 0.0001. Scale bars: 50 μm. Deadpan data is adapted from [24].

Key transcription factors such as Nervous fingers-1 (Nerfin-1), Midlife Crisis (Mdlc), and Longitudinal lacking (Lola) were shown by us and others to maintain neuronal identity in *Drosophila* [9,10,21,22]. In the medulla, the *Drosophila* visual processing centre, we previously showed that Nerfin-1 maintains neuronal fate in conjunction with its co-factor the Hippo pathway transcription factor, Scalloped (Sd) [22]. Interestingly, the vertebrate ortholog of Nerfin-1, Insulinoma-associated 1a (Insm1a), is a master regulator of zebrafish retinal regeneration, playing roles in multipotency and cell cycle exit [23]. Our targeted DNA adenine methyltransferase identification (DamID) analysis in the *Drosophila* medulla neurons identified 3587 target genes commonly regulated by Nerfin-1 and Sd [22]. 470 candidate Sd/Nerfin-1 target genes were upregulated in the neural stem cells (neuroblasts, NBs) compared to neurons, while 656 genes were downregulated. 36 of these genes encode transcription factors, of which, 12 were expressed in either NBs or neurons. To assess if any of these genes were functionally involved in dedifferentiation, we used a heat shock-induced *actin-GAL4 flp-out* (*hs flp*) (Fig 1D), to induce clones that either overexpress or knockdown candidate genes. RNAis were used to knockdown the expression of these genes if they were expressed in neurons, and UASs were used to overexpress the candidate genes expressed in NBs. Additionally, we used a medulla driver (*GMRH108-GAL4*) to validate our findings (the result of our dedifferentiation screen is summarised in S1 Table).

As medulla NBs are only observed in the superficial layer and neurons in the deep layers, we scored for dedifferentiation by assessing the presence of the NB marker, Miranda (Mira), in the deep neuronal layers of the medulla (Fig 1A). We hypothesised that candidate transcription factors specifically expressed in the neurons would act as transcriptional repressors to silence the neural stem cell fate. Therefore, downregulating these genes in the neurons, should yield ectopic NBs, indicative of dedifferentiation (Fig 1B and 1C). Conversely, transcription factors expressed in the NBs would act as transcriptional activators and must be silenced in the neurons to maintain differentiation. Overexpressing these factors in the neurons would produce ectopic NBs. Upon induction of the bHLH transcription factor, Deadpan (Dpn), we observed ectopic NBs (as indicated by Mira+ cells) in the medulla neuronal layer of the larval CNS compared to control clones (Fig 1E–1F’ and 1J). Similarly, knockdown of the homeobox transcription factor, Prospero (Pros) and high mobility group box (HMGB) transcription factor, SoxNeuro (SoxN), induced numerous ectopic Mira+ cells in the deep layer (Fig 1H–1I’ and 1J). Interestingly, knockdown of Embryonic lethal abnormal vision (Elav) resulted in the formation of ectopic NBs only in a subset of medulla neurons (Fig 1G–1G’, arrows and 1J). Therefore, Elav may only be required to maintain the identity of a subset of neurons in a temporally restricted fashion. Together, this data shows that overexpression of Dpn and knockdown of Pros, Elav, and SoxN phenocopied Nerfin-1 loss of function, and thus are likely mediators of dedifferentiation. As factors implicated in dedifferentiation may be involved in the regenerative response upon injury, we next examined whether these factors are involved in zebrafish retina regeneration.

### Characterisation of MTZ-induced adult photoreceptor injury model

To study the process of regeneration in adult zebrafish, we used a metronidazole (MTZ) inducible transgenic fish line to specifically ablate the highly abundant long wavelength sensitive (lws) photoreceptors (PRs). Following standard protocols [5], we subjected adult fish (11 months of age) to MTZ for 24 hours, followed by a 72-hour recovery time. We observed PR reduction (mCherry-labelled cells) at 72 hours post ablation (hpa) (S1A–S1B”’ Fig, arrows), suggesting MTZ-induced injury was effective and, therefore, for the rest of the experiments, fish between 10 and 12 months were utilized. Additionally, we characterised the re-appearance of mCherry within newly regenerated photoreceptors. As the lws2 (opsin) promoter drives mCherry expression in mature PRs, we looked at a variety of later time points following regeneration, i.e., at 7-, 9-, and 12-days post ablation (dpa) (S1C–S1D” Fig). We found that mCherry expression did not return until 12dpa. Thus, we needed a marker to assess PR regeneration at earlier timepoints, and therefore used zpr-1, which labels Arrestin 3 (Arr3) expressed in red and green cones [25], to examine cone regeneration.

Next, we conducted a time course experiment at 16, 24, 48, 72, and 96 hpa to characterise the regenerative response under our ablation paradigm. Müller glia (MG), located in the inner nuclear layer (INL) and labelled by Glutamine Synthase (GS), are known to produce progenitor cells, labelled by Proliferating Cell Nuclear Antigen (PCNA) upon injury (S3A–S3G”’ Fig). Consistent with other adult injury paradigms [26], MG-derived progenitors were first observed at around 24hpa (S3D” Fig, outlined). They began proliferating at 48hpa, as indicated by clusters of PCNA+ cells across the INL (S3E” Fig, outlined), also observed by at 72 and 96 hpa (S3F”and S3G” Fig, outlined). This increase in PCNA+ cells was accompanied by a more diffuse GS staining pattern at 72 and 96hpa, consistent with the dilution of MG markers into the neuronal progenitors (S3F”’ and S3G”’ Fig, outlined). The regenerative response observed in our MTZ induced injury model is comparable to that reported for other PR injury paradigms [26], therefore, our MTZ model of degeneration is consistent with other previously described damage models.

### Her6/Hes1 is expressed at lower levels in proliferative MG-derived progenitors

As the *Drosophila* genes *deadpan* (*dpn*) and *prospero* (*pros*) induced dedifferentiation (S1 Table) and their homologs *hairy-related 6* (*her6*) and *prospero homeobox 1a* (*prox1a*) have not previously been implicated in PR regeneration, we next set out to assess the function of these novel regulators utilising our MTZ-mediated injury model.

**Fig 2:**
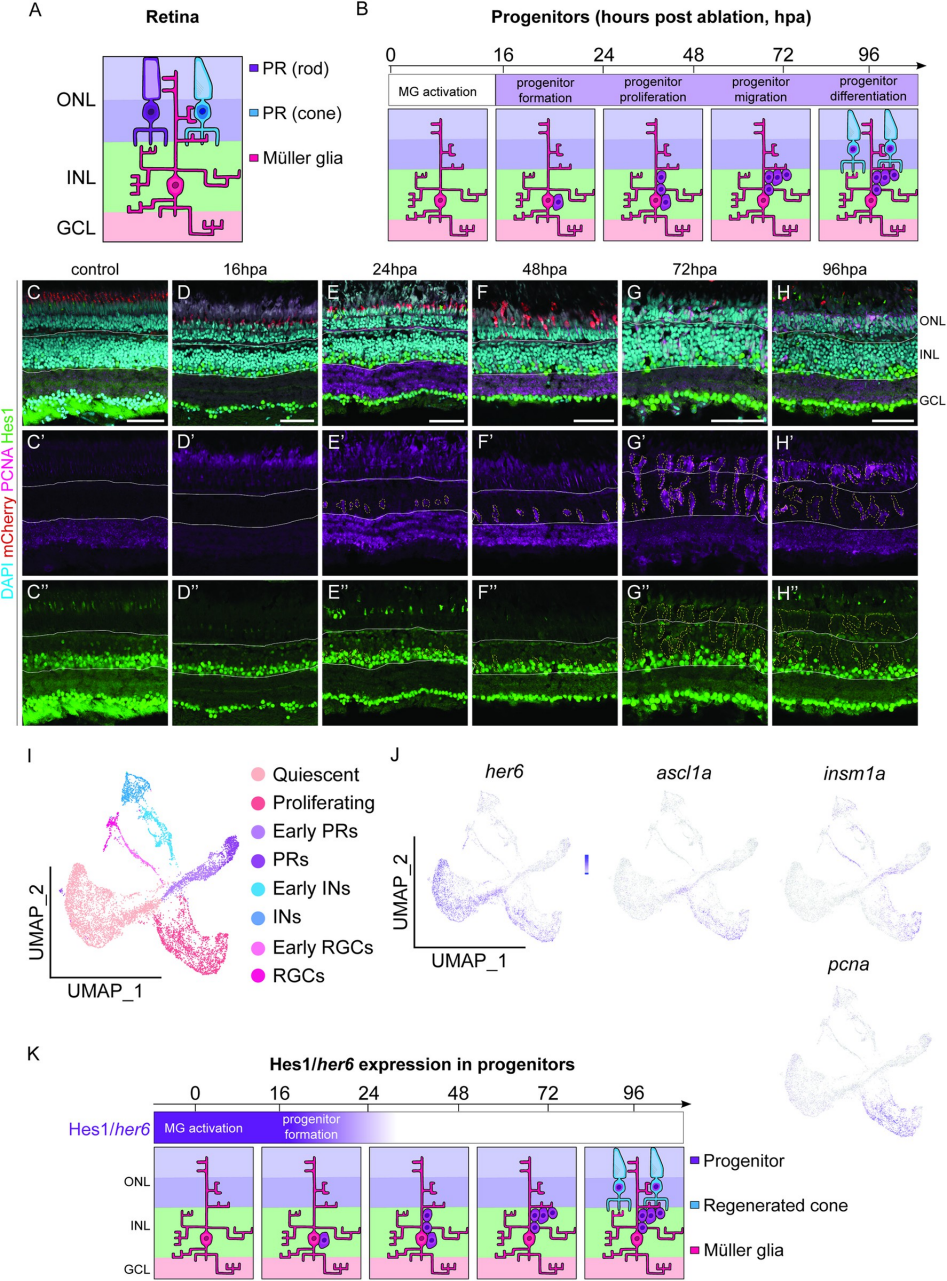
Her6/Hes1 is reduced in regenerating progenitors. (A, B) Schematic representation of retinal cells and observed regenerative processes and timeline. Rod (purple) and cone (blue) photoreceptors (PR) are located in the outer nuclear layer (ONL). Müller glia (MG) are located in the inner nuclear layer (INL) with processes spanning across the retina from the ONL through to the GCL (ganglion cell layer). Following PR ablation, MG are activated and dedifferentiate into progenitors, which proliferate and migrate prior to differentiation into photoreceptors by 96 hours post ablation (hpa). (C-H”) In adult retina, during the 96-hour regenerative time span following the ablation of PRs (red), proliferation is marked by proliferative cell nuclear antigen (PCNA, pink) starts at 24 hpa. Expression of Hairy-enhancer of split (Hes1, green) is seen both in the control and following the ablation in the inner half of the INL and in the GCL. Focusing on the proliferative cells, the expression of Hes1 is downregulated as PCNA expression increases from 24 hpa onwards. DAPI (cyan) marks the retinal layers. Scale bars: 50 μm.(I) UMAP plot of FACS MG 3 days after injury is subdivided into distinct MG derived cell populations based on stereotypical markers. (J) The feature plots show that *her6* is expressed mainly in the quiescent and some proliferating MG (*pcna*, *ascl1a* expressing) subsets and downregulated in differentiating (*pcna*, *insm1a* expressing) cell populations. (K) Summary of Hes1/*her6* expression during regeneration. Hes1/*her6* is present in the initial progenitors and downregulated upon their proliferation.

Dpn is expressed in NBs and necessary for the maintenance of NB ‘stemness’ (San-Juán annbd Baonza 2011), and more recently, we have shown that Dpn overexpression is sufficient to induce neuron to NB dedifferentiation [24]. The zebrafish ortholog of *dpn*, *her6*, is expressed in developmental progenitors within the inner nuclear layer (INL) and is downregulated upon neurogenesis [27]. To investigate whether Her6 played a role during regeneration, we assessed its expression in a time course analysis upon PR injury, between 16 and 96hpa (Figs 2A, 2B and S3H). We utilised an antibody against the murine ortholog of Her6, Hairy and enhancer of split-1 (Hes1), however, this may in addition to Her6, may label other members of the Her family. We found that Hes1 was expressed in the INL (Fig 2C–2C”, outlined) in the control retina. Upon injury, between 16 and 24hpa, Hes1 expression appeared downregulated, and by 48hpa Hes1 was no longer detected within the PCNA+ progenitor cells (Fig 2D’–2H”). Additionally, we performed a time course study, to examine the colocalization between Hes1 and MG cells (marked by GS). We found that between 16 and 24hpa, Hes1 is expressed within the MG, however, from 48hpa onwards, there is little Hes1 expression within GS-positive cells (S4A–S4F”’ Fig). This suggests that Hes1 may be downregulated upon progenitor formation and proliferation.

To further assess *her6* expression, we turned to a MG single-cell RNA sequencing (sc-RNAseq) dataset generated in the larval zebrafish at 72hpa [17]. We found that the regenerative profile of the larval zebrafish was comparable to that of our adult injury model (S4 Fig), and that MG-derived progenitors first appeared by 24-48hpa and were labelled by PCNA (a proliferative marker) at 72hpa (S4 Fig). As larval and adult regenerative response during injury was comparable, we then assessed the expression of *her6* in the larval MTZ-induced injury model sc-RNAseq dataset. Through unbiased UMAP clustering, we matched differing MG cell states using key known markers [17]. These consisted of quiescent and activated MG as well as proliferating neuronal progenitors and differentiating MG-derived progenitor cells: early PRs, PRs, early inhibitory neurons (INs), INs, early retinal ganglion cells (RGCs), and RGCs (Fig 2I). Within the *pcna*-expressing proliferative cluster, we found that *her6* was highly expressed within cells in the reprogrammed MG (defined by *ascl1a* expression, [7]) and was downregulated in the proliferating and differentiating populations of neuronal progenitors (defined by *insm1a* expression, [7]) (Fig 2J). This is consistent with our Hes1 time-course analysis in the adult injury model, where we observed a downregulation of Hes1in the PCNA+ MG progenitor cells at later stages of proliferation (Fig 2K). Together, our data suggest that Her6/Hes1 may act as in inhibitory regulator, and its downregulation may be required for regeneration to occur.

### Knockdown of *her6* results in an increase of PRs after injury

**Fig 3:**
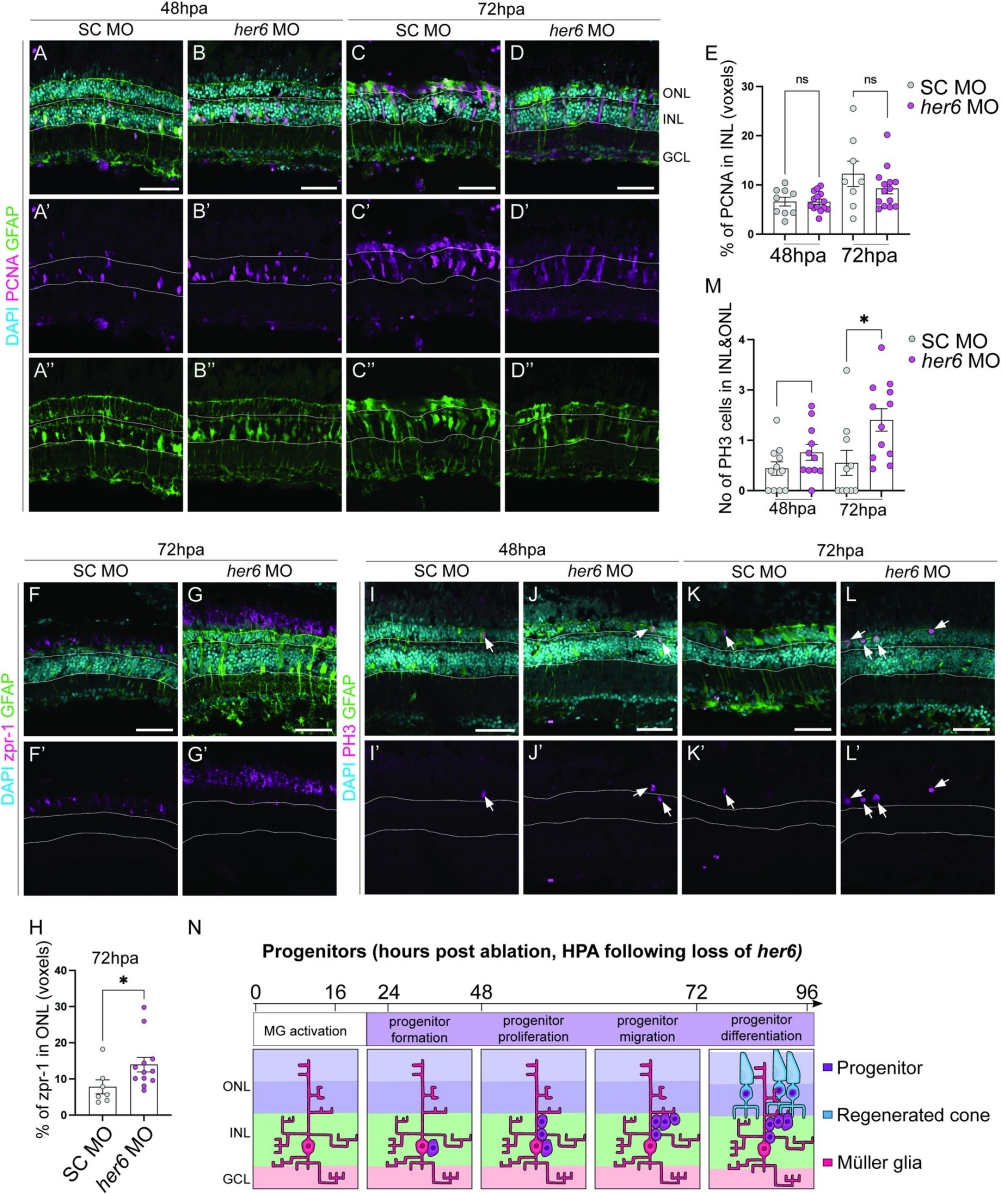
Loss of Her6 increases PR production at 72 hours post ablation. (A-D”) In adult retina, at 48 and 72 hours post ablation (hpa), the number of proliferating cells marked by PCNA (pink) and Müller glia (MG) cells marked by glial fibrillary acidic protein transgene expressing (GFAP, green) are not significantly altered between standard control (SC, 48hpa n = 9, 6.621 ±0.9034, 72hpa n = 8, m = 12.27 ±2.579) and *her6* morpholino (MO, 48 hpa n = 14, m = 6.59 ±0.5219, 72 hpa n = 14, m = 9.299 ±1.1) electroporated samples quantified in (E). (F-G’) In *her6* MO samples, there was a significantly increase in zpr1 (pink, n = 12, 13.97 ±2.202) labelled cone photoreceptors compared to SC (n = 7, 7.809 ±1.938), quantified in H. (I-L) There was also an increase in phospho-histone 3 (PH3, pink) marking mitotic cells in the *her6* MO (48hpa, n = 11, 1.519 ±0.3166, 72hpa n = 12, 2.806 ±0.4442) compared to SC (48hpa n = 11, 0.8812 ±0.2671, 72hpa (SC n = 10, 1.102 ±0.4956) quantified in M. In all micrographs DAPI is used to label nuclei cyan. Data are represented as mean ± SEM. *p < 0.05. Scale bars: 50 μm. (N) Summary of regenerative phases and timeline in *her6* MO electroporated fish showing an increase in mature photoreceptor regeneration.

To further explore Her6 during regeneration, we assessed whether it was functionally required. We knocked down *her6* expression via *in vivo* electroporation of morpholino (MO) within the adult retina [28]. This method allowed us to assess the role of developmentally required genes in the context of adult injury. First, we assessed the efficiency of *her6* knockdown via Hes1 antibody staining. We found that Hes1 levels was reduced, though not significantly, as Hes1 antibody may label other members of the Hes/Her family (S6E–S6I Fig). Fish were subject to 24-hour MTZ treatment to ablate long wavelength sensitive (red) cones immediately following MO injection and electroporation. We observed an overall increase of the number of red / green cones, indicated by zpr-1 at 72hpa (Fig 3F–3G’ and 3H), however, this was not accounted for by an increase in the number of PCNA^+^ progenitors at 48 and 72hpa, compared to the standard control (SC) MO (Fig 3A–3E). Additionally, it was not accounted for by the protective effects of *her6* MO electroporation, as we observed efficient cell ablation under these manipulations (S6A–S6C” Fig). To assess whether the rate at which the progenitor cells underwent proliferation, we used Phospho-Histone3 (PH3) to mark cells cycling through M-phase of the cell cycle. Interestingly, we observed a higher number of PH3+ cells in the *her6* MO-treated fish at 72hpa, compared to control (Fig 3I–3M). This suggests that the loss of Her6 may increases the speed at which progenitors progress through the cell cycle, however, it could also indicate a prolonged mitotic phase. Together, our data suggest that Her6 is a negative regulator of regeneration, and its downregulation promotes cone production upon injury (Fig 3N).

### Prox1a is expressed in differentiating PRs after injury

Next, we assessed whether the homolog of *Drosophila* Prospero (Pros), Prospero homeobox1a (Prox1a), was involved in zebrafish retina regeneration. *Drosophila* Pros is required for cell cycle exit in NBs, and its loss results in the formation of ectopic NBs [29]. Prox1a is involved in the differentiation of inhibitory neurons (amacrine and horizontal cells) within the developing mouse retina [30], however, the role of Prox1a in retinal regeneration is so far unknown.

**Fig 4:**
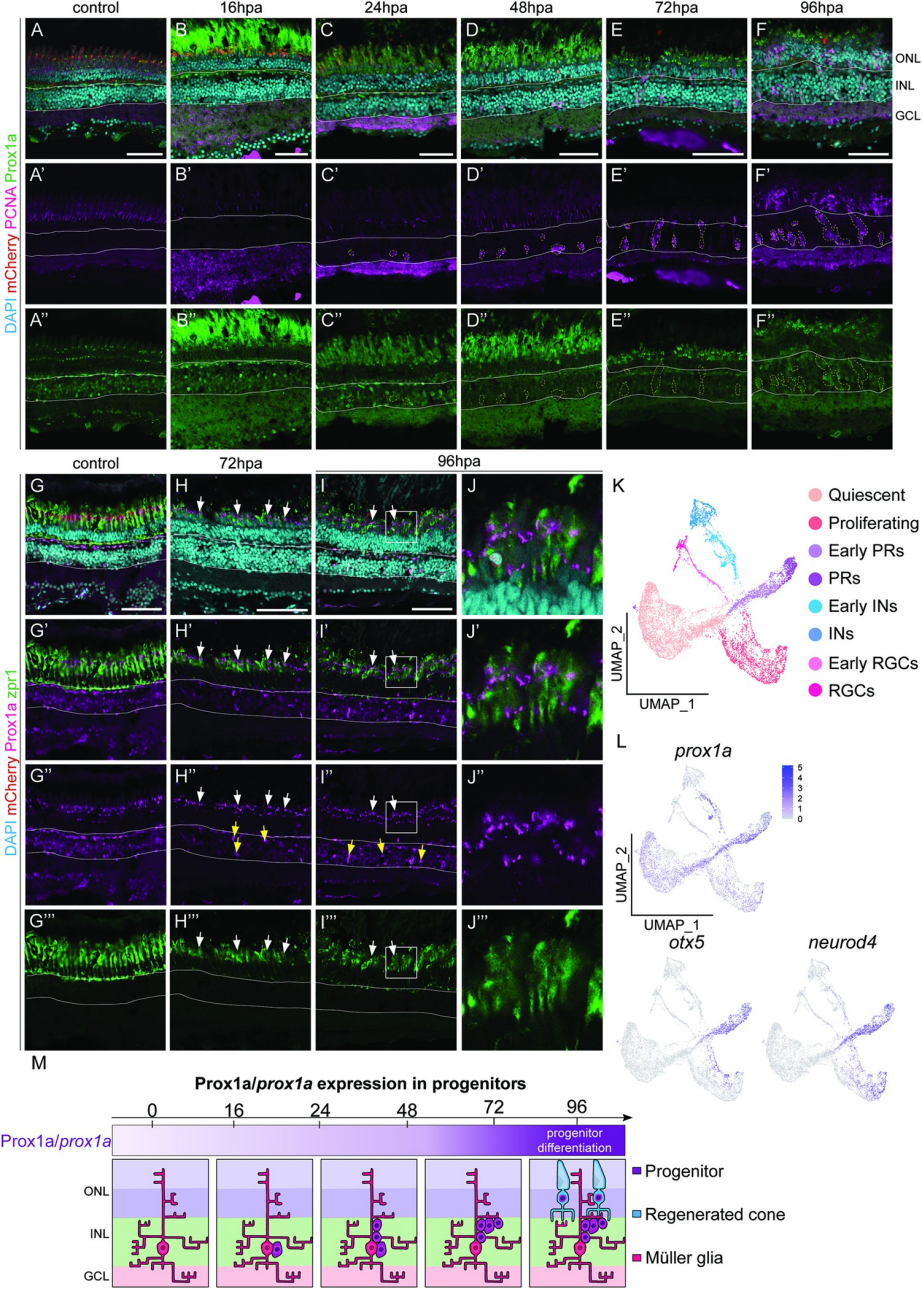
Prox1a is expressed in the photoreceptors within the outer nuclear layer. (A-F”) Within adult retinas in uninjured control, post ablation (hpa), Prospero homeobox1a (Prox1a, green) is expressed in the inner nuclear layer (solid white outline), but never in proliferating (proliferative cell nuclear antigen positive, PCNA, pink) cells (dotted outlines). At 72hpa and 96hpa Prox1a in addition expressed in the ONL. (G-I”) cone photoreceptor marker, zpr1 (green) is expressed in the ONL in the control and at all the time points post ablation. (J-J”’) is the magnified images of (I-I”’). At 72hpa and 96hpa, Prox1a is expressed as distinct puncta within zpr1 expressing photoreceptors (arrows and high-power inset). Scale bars: 50 μm. (K, L) UMAP plot of FACS MG 3 days after injury can be subdivided into distinct MG derived cell population based on stereotypical markers. The feature plots show that *prox1a* is strongly expressed in the *neurod4* and *otx5* positive early photoreceptors. (M) Summary of Prox1a/*prox1a* expression during regeneration.

To assess how Prox1a may be involved in regeneration, we performed a time course analysis to assess Prox1a expression during regeneration. We found that Prox1a was consistently expressed within the INL of control retinas (Fig 4A–4F”). Its expression was not altered in the MG-derived progenitors upon injury (Fig 4C”–4F”, outlined). However, Prox1a was upregulated in the differentiating PRs from 72hpa (Fig 4E” and 4F”).

Consistent with this, we identified prominent *prox1a* expression within the differentiating PR population in our sc-RNAseq dataset, as *prox1a* was highly upregulated in the cell population where PR-specific genes, *neurod4* and *otx5* were also upregulated (Fig 4K–4M). To assess this qualitatively, we conducted an additional time course and studied Prox1a expression along with the red/green cone PR-specific marker, zpr-1. Indeed, we found that Prox1a was expressed within zpr-1 expressing cells at 72 and 96hpa, the time point at which PRs undergo differentiation (Fig 4G–4J”’ arrows). As Prox1a is expressed in differentiating PRs (Fig 4M), it may be required to promote PR differentiation after injury.

### *Prox1a* knockdown results in a reduction of PR production after injury

**Fig 5:**
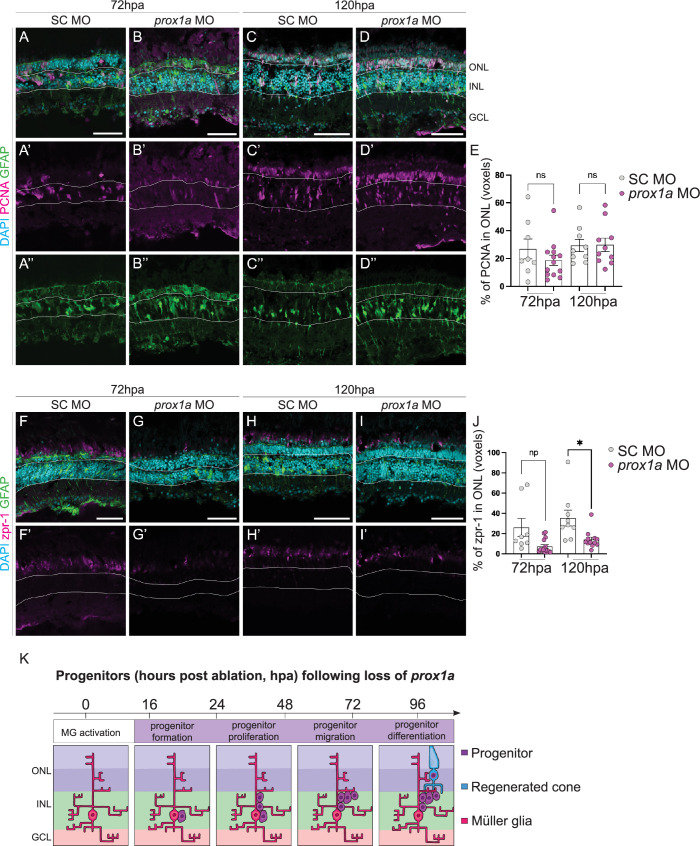
Loss of Prox1a decreases PR production at 120 hours post-ablation. (A-D”) Knockdown of Prox1a via morpholino (MO) in adult retina during regeneration does not significantly affect percentage of PCNA+ (pink) proliferative Müller glia (marked by GFAP, green) following *prox1a* MO (72hpa n = 13, 18.7 ±03.692, 120hpa n = 10, 29.84 ±4.805) compared to SC (72hpa n = 8, 26.82 ±7.14, 120 hpa n = 9, 29.37 ±4.408), quantified in E. (F-I’) Knockdown of Prox1a resulted in a significant reduction of cone photoreceptors as marked by zpr1 (72hpa n = 15, 7.312 ±1.806, 120hpa n = 12, 14.05 ±2.539) compared to SC (72hpa n = 8, 25.98 ±8.962, 120 hpa n = 9, 35.4 ±7.836) in the ONL, quantified in J. DAPI labels cell nuclei (cyan). Data are represented as mean ± SEM. *p < 0.05. Scale bars: 50 μm. (K) Summary of regenerative time course following Prox1 MO knockdown showing reduction in mature regenerated photoreceptors.

To assess the function of Prox1a in PR regeneration, we knocked Prox1a down using *in vivo* MO electroporation (the efficiency of the knockdown is shown in S5A–5D”’ and S5I Fig). We found at 72 and 120hpa, there was no significant change in the percentage of PCNA+ cells (Fig 5A–5E), suggesting Prox1a does not regulate progenitor formation or proliferation. However, there was a significant decrease in the percentage of cells expressing the mature PR marker, zpr-1, by 120hpa (Fig 5F–5J). Our data suggests that Prox1a is required for PR differentiation following injury, in its absence, differentiated mature PRs fail to be produced (Fig 5K). Using PH3, we observed an unexpected increase in mitotic cells at 72 hpa but not at 120 hpa (S7D–S7H Fig), possibly reflecting changes in cell cycle dynamics.

### Prox1a may act through LLPS in PR differentiation

**Fig 6:**
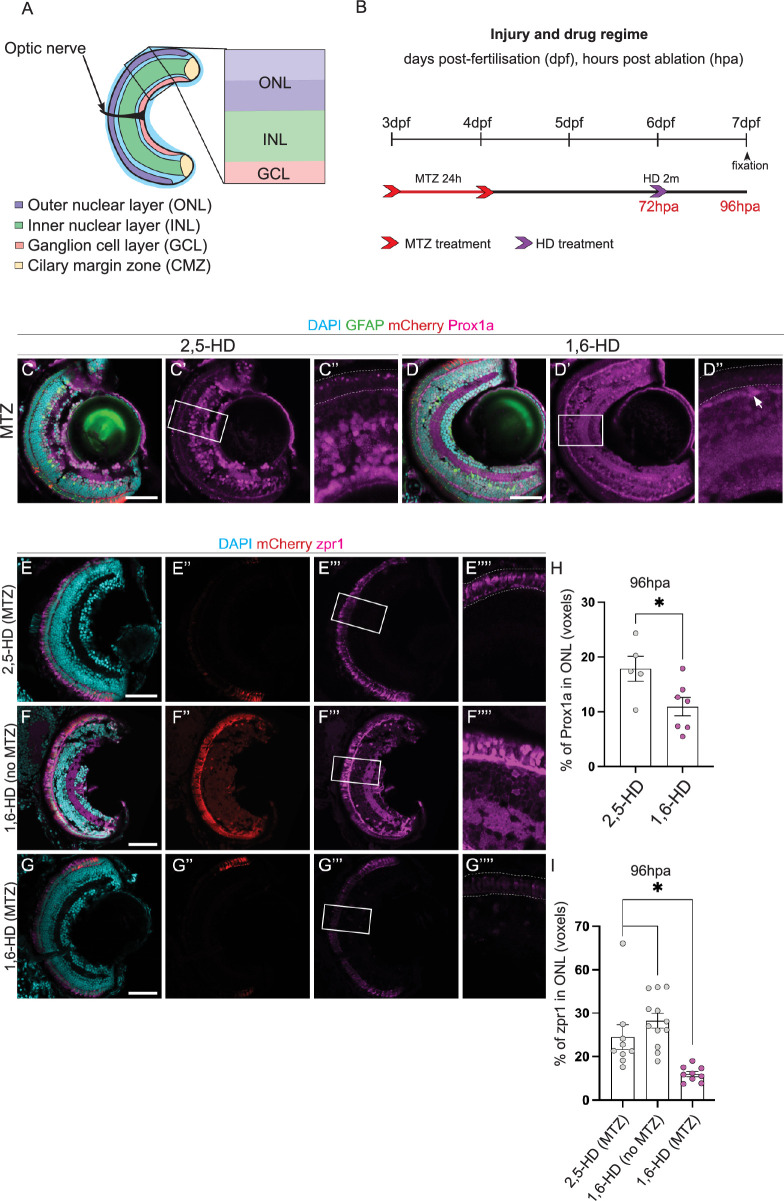
Prox1a puncta in liquid-liquid phase separates regulate PR differentiation. (A) Schematic highlighting the retinal layers analysed in the zebrafish larvae. (B) Experimental timeline. Larvae were swum in metronidazole (MTZ) for 24 hours at 3dpf (days post-fertilisation), followed by a 2 min treatment with 1,6-Hexanediol (1,6-HD) or 2,5-HD at 6dpf, before processing at 7dpf. (C-D”) Following photoreceptor (PR) ablation, Tg(*lws2*:*nfsb-mCherry*, *gfap*:*eGFP*) retinas show Prospero homeobox1a (Prox1a, pink) puncta only in the ONL of 2,5-HD control treated retinas (n = 5, 17.86 ±2.3), but not when liquid-liquid phase separates have been disassembled in the 1,6-HD treated samples (n = 7, 10.94 ±1.681, quantified in H. Arrow indicates nuclear Prox1a staining outside of the photoreceptor layer. (E-G”’) zpr1 cone photoreceptors are observed in both control samples (2,5-HD treated ablated retinas and 1,6-HD treated, non-ablated retinas), but much reduced in the ONL of 1,6-HD treated ablated retinas, quantified in I. DAPI labels cell nuclei (blue). Scale bars: 50 μm.

We next assessed the role of Prox1a in PR differentiation after injury. Interestingly, throughout our investigations Prox1a protein appeared in small puncta within the PRs (Fig 4J”). This was reminiscent of the protein puncta the *Drosophila* ortholog of Prox1a, Pros, forms during differentiation [31], where Pros was shown to regulate neuronal differentiation though Liquid-liquid Phase Separation (LLPS). We next assessed whether Prox1a may drive PR differentiation during regeneration in a similar fashion. Many publications, including those using zebrafish models, assessed LLPS through 1,6-Hexanediol (1,6-HD) treatment [32]. 1,6-HD is an organic compound commonly used to dissolve liquid-liquid phase separates [33]. However, this has only been carried out *in vitro*. As it is more challenging to perform high throughput analysis in adult fish, we switched to the larval injury model (Fig 6A) to first determine at which concentration 1,6-HD and the control 2,5-HD (a drug which is structurally very similar to 1,6-HD but with a much lower droplet melting activity [34]) can be administered to zebrafish. Through trialling a variety of concentrations, exposure times, and recovery times, we found that the lowest treatment that was most effective and consistent was administration of 5% for 2 minutes (S7–S9 Figs). Therefore, at 3dpf, we swam larvae in MTZ for 24 hours, allowed them to recover for 72 hours, then they were treated with 5% of either 1,6-HD or 2,5-HD for 2 minutes, and fixed and stained 24 hours later (at 96hpa) (Fig 6B). Overall, we found significantly less Prox1a in the ONL in 1,6-HD treated larvae compared to 2,5-HD treated larvae (Fig 6C–6D” and 6H), suggesting that inhibiting phase separation may disrupt Prox1a levels. Additionally, the presence of Prox1a in cell types within the INL acted as an internal control, as there was normal diffuse Prox1a expression remained in these cells (Fig 4H” and 4I”, yellow arrows). To assess whether phase separated Prox1a was functionally required for the regenerative response after injury, we examined zpr-1 expression in 1,6-HD treated larvae. We discovered that there was significantly less zpr-1 within the ONL in 1,6-HD treated larvae compared to control (Fig 6E–6E”“, 6G–6G”“and 6I). Additionally, we show that there is no difference in zpr-1 expression within no-injury 1,6-HD treated larvae compared to injured 2,5-HD treated larvae (Fig 6F–6G”“and 6I), demonstrating that 1,6-HD treatment alone does not affect PR production. Together, our data suggest that Prox1a may form liquid-liquid phase condensates within PRs and this process may be necessary for PR differentiation after injury. However, we acknowledge that these experiments are exploratory and more in-depth research must be undertaken to understand the underlying mechanisms.

### *her6* knockdown increases Prox1a expression

**Fig 7:**
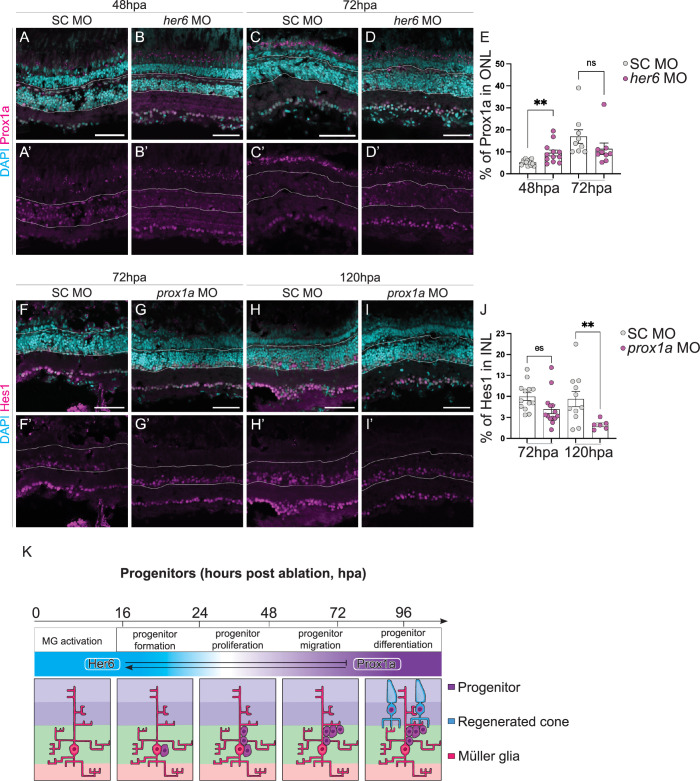
Her6 reduction increases Prox1a expression. (A-D’) Downregulation of *hairy-related 6* (*her6*) by morpholino (MO) in adult retina resulted in a significant increase of Prospero homeobox 1a (Prox1a) in the ONL during early phases of regeneration at 48hpa (SC n = 15, 5.294 ±0.3032, *her6* MO n = 12, 9.507±1.339), but not at 72hpa (SC n = 9, 16.97 ±3.096, *her6* MO n = 9, 11.34 ±2.625), quantified in E. (F-I’) Knockdown of Prox1a using MO led to a significant downregulation of Hes1/Her6 expression at 120 hpa (SC n = 11, 9.355 ±1.792, *prox1a* MO n = 6, 3.255 ±0.4484), but not at 72 hpa (SC n = 13, 9.958 ±0.9212, *prox1a* MO n = 14, 6.929±1.044), quantified in J. (K) Summary of expression and cross-regulation between Her6 and Prox1a expression during regeneration. Her6 is expressed during progenitor formation while Prox1a is present in differentiating PRs. Her6 is a negative regulator of Prox1a and Prox1a is a positive regulator of Her6. Data are represented as mean ± SEM. *p < 0.05. Scale bars: 50 μm.

As Her6 and Prox1a knockdowns both affected PR production during regeneration, it is interesting to speculate whether they function in the same pathway. DNA adenine methyltransferase IDentification (DamID) analysis, a tool mapping binding sites of chromatin binding proteins, previously showed the *Drosophila* ortholog of *prox1a*, *pros*, is a target gene of the *her6* ortholog, *dpn* [35]. Additionally, Dpn acts in neural stem cells to repress Pros, and its loss results in increased Pros expression and differentiation [36]. In the regeneration context, we showed that Her6 knockdown resulted in enhanced progenitor proliferation and additional PRs production, while Prox1a regulated PR differentiation. To examine whether Her6 and Prox1a intersect, we assessed Prox1a expression following Her6 knockdown via MO. Interestingly, at 48hpa, a very early time point in the regenerative process, we observed significantly more Prox1a expression compared to control (Fig 7A–7E), suggesting that the loss of Her6 may relieve the suppression of Prox1a during early stages of regeneration, to promote precocious PR generation. However, by 72hpa, the number of Prox1a cells was no longer significantly different between *her6* MO and control. Together, this data suggests that Prox1a may lie downstream of Her6 and is negatively regulated by Her6 (Fig 7K).

Finally, we examined whether Her6 is also regulated by Prox1a. Upon knockdown of Prox1a, we found that Hes1 levels were significantly reduced (Fig 7F–7J). Therefore, in addition to the role of Prox1a in regulating PR differentiation, Prox1a is required to positively promote Her6 levels (Fig 7K).

Together, through examining the role of factors implicated in dedifferentiation in *Drosophila* optic lobes, we have identified two novel regulators of zebrafish retinal regeneration. Her6 may negatively inhibit cell-cycle progression of MG-derived progenitors and PR production, while Prox1a mainly regulates later stages of PR differentiation, to promote the overall production of zpr-1+ PR cells.

## Discussion

In this study, we identified two evolutionary highly conserved novel candidates of regeneration, which may be used in pro-regenerative settings to improve the human regenerative response following injury. Indeed, many of the genes already identified to positively regulate PR regeneration in zebrafish were shown to improve neural regeneration in the mammalian retina [37–39]. This highlights the necessity and utility of such cross-species research.

### Her6 in regeneration

We found that Her6 is downregulated upon progenitor formation and proliferation during regeneration; furthermore, downregulation of Her6 increased PR production. Whether these PRs mature faster or there is additional PR production remains unknown. We did not find that there was an increase in progenitors; while PH3 analysis indicated that more cells cycled through M phase, additional cell cycle analysis (e.g., S phase marker BrdU), is required for us to conclude whether there was enhanced proliferation of the progenitors. In addition, there may be dynamic changes at different stages of regeneration, this can be addressed by performing a more refined time course analysis. Additionally, assessing the specific PR subtype that are altered during Her6 knockdown would allow us to identify whether Her6 acts in specific PRs. The significant increase in PRs, together with the expression profile of Her6 during regeneration, indicates that Her6 is a negative regulator of regeneration, which may play important roles in early progenitor cells to regulate regeneration. Finally, Her6 may be repressing genes involved in proliferation and differentiation. An example of this may be Prox1a, as we found that a decrease of Her6 expression negatively inhibited PR production and the onset of Prox1a.

It was surprising to see that manipulating Her6 had the potential to further enhance regeneration, even in a regenerative model such as the zebrafish. We found that Her6 is downregulated upon progenitor formation and proliferation; knocking down Her6 was able to further increased PR production. Moreover, Her6 downregulation may be required for MG activation and proliferation. Her6 has previously been shown to be expressed in the MG and progenitor cells of the CMZ [40]. *her6* and its human ortholog, *hairy and enhancer of split 1* (*hes1*), are well-defined Notch targets [41–43]. Interestingly, upon the knockdown of Notch-Delta activity, Her6 expression was lost [40]. During retinal regeneration, similar to Her6, Notch signalling is downregulated to enable Müller glia (MG) to enter the cell cycle [44,45]. Additionally, the loss of Notch signalling is sufficient to induce MG proliferation in the undamaged retina [46]. In future studies, it would be interesting to test whether the loss of Her6 can stimulate quiescent MG to re-enter cell cycle in the absence injury. *Drosophila* ortholog of *her6*, *deadpan* (*dpn*), is also a Notch target gene, however, it plays an opposite function within neurons, where the overexpression of Dpn triggers the transition of mature neurons to NSCs [24]. Together, this highlights the dynamic roles of Notch signalling in different cell types.

Notch signalling is known for acting as both positive and negative regulators in many biological processes. RT-PCR and sc-RNAseq identified that the Notch receptor, Notch3, and ligand, DeltaB, were downregulated upon retinal damage [47]. Additionally, their individual losses were sufficient to induce the entry of quiescent MG into the cell-cycle [47], phenocopying previously studied roles of Notch in the zebrafish retina. We suggest that Her6 likely lies downstream of Notch3 due to their similar phenotypes.

### Prox1a in regeneration

Even though the function of Prox1a is well established within INL progenitors [30], here, for the first time, we demonstrate a role for Prox1a in PR differentiation specifically during regeneration. We found that Prox1a was expressed in and promoted the differentiation of progenitors following PR loss. Strikingly, we observed the punctate staining pattern observed in the ONL, in contrast to the nuclear localisation observed in other neurons throughout the retina. We suggest that this may occur through Liquid-liquid phase separation (LLPS). We assessed LLPS involvement through the treatment of 1,6-hexanediol (1,6-HD). This compound dissolves phase-separates and is commonly used to determine the role of LLPS, including in a recent study involving the *Drosophila* ortholog of *prox1a*, *prospero* (*pros*) [31]. While additional assays, such as temperature increases, could also be used to destabilise phase-separates [48,49], our results clearly show that disruption of LLPS led to significantly less Prox1a expression in the outer nuclear layer (ONL) following 1,6-HD treatment, without affecting Prox1a within the same samples in the INL.

### Morpholinos as an adult system for protein knockdown

MOs have been broadly used in developmental biology to assess the role of genes in loss of function scenarios. Whilst not as efficient or long-lasting as stable mutant lines, MO use also knockdowns maternally deposited RNA, and, furthermore, circumvents the issue of genetic compensation observed with some mutants [50]. For adult regenerative studies, MOs provided a great tool to assess differences in a regenerative phenotype upon gene knockdown. Specifically, we could assess adult regeneration without interfering with the important function many of the involved genes play during development, thus providing targeted, inducible and tissue-specific knockdown of gene function. Limitations that are important to keep in mind are efficiency and specificity issues [51], and the fact that in our study both the metronidazole induced PR ablation, as well as the process of MO electroporation, may contribute to retinal damage that triggered the regenerative process. Our study demonstrates that even with only partial and mosaic knockdown, clear phenotypic differences can be observed following *her6* and *prox1a* MO, further emphasising their critical role in regeneration.

### Complementary—model systems for identifying regeneration regulators

Through a candidate-based across species approach, we identified novel regulators of regeneration. Firstly, we utilised the high-throughput system of *Drosophila* to conduct a genetic screen, searching for novel regulators of dedifferentiation (a cell fate process with key similarities to regeneration), downstream of the dedifferentiation regulator, Nervous fingers-1 (Nerfin-1). Interestingly, the vertebrate ortholog of Nerfin-1, Insulinoma-associated 1a (Insm1a), has a dual function in zebrafish retinal regeneration, playing roles in both multipotency and cell-cycle exit. Upon activation via Achaete-scute complex like 1a (Ascl1a), Insm1a can function in a positive feedback-loop to increase Ascl1a expression, to activate multipotency and proliferation factors [23]. However, Insm1a is also essential for cell cycle exit by repressing BAF chromatin remodelling complex subunit (Bcl11) which in turn stimulates the expression of the cyclin-dependant-kinase (CDK) inhibitor: p57kip2 [23]. Additionally, other targets of Nerfin-1 have known roles in zebrafish retinal regeneration. We showed that the downregulation of the Nerfin-1 target, SoxNeuro (SoxN), in the medulla neurons induced ectopic stem cells. The zebrafish ortholog of *SoxN*, *sry* (*sex determining region Y*)-*box 2* (*sox2*), has a well-established role during regeneration. Sox2 is a multipotency factor required for the reprogramming of activated MG and its loss during regeneration decreases the proliferation of MG-derived progenitors [52]. Interestingly, this occurs through the activation of Ascl1a, whose *Drosophila* ortholog, *asense* (*ase*), is also a target of Nerfin-1. Together, this highlights the similarities between the genes and networks involved in dedifferentiation and regeneration as well as their conservation across model species. These similarities can be exploited to identify new factors involved in vertebrate regeneration with the potential to increase regeneration in humans.

## Supporting information

S1 Table Genetic screen for candidates involved in dedifferentiation. The name of the gene, location of expression, types of gene manipulation (overexpression (OE) / RNAi) and result of screen under both *GMRH108Gal4* and *hs FLIP actin* promoters are shown. Grey indicates the candidate genes that were screened. Green indicates that Miranda positive ectopic stem cells were identified. The misexpression of *deadpan* and *prospero* induced Miranda positive cells under both promoters. The knockdown of *SoxNeuro* and *elav* induced Miranda positive cells under the *hsFLIP actin* promoter.
(PDF)

S1 Fig Photoreceptors are ablated using MTZ treatment in adult fish. (A-B”’) Metronidazole (MTZ) induced ablation of mCherry labelled photoreceptors in Tg(*lws2*:*nfsb-mCherry*, *gfap*:*eGFP*) adult zebrafish efficiently ablated the targeted cells as shown at 72 hours post ablation (hpa). (C-D”) Expression of the lws2 promoter driven mCherry in regenerated cells was re-established by 12 days post ablation (dpa). DAPI labels nuclei (cyan) and Glial fibrillary acidic protein (GFAP, green) marks Müller glia. Scale bars: 50 μm.
(PDF)

S2 Fig Müller glia derived progenitors drive regeneration starting 24 hours post-injury in the adult zebrafish. (A) Summary of adult retinal regeneration stages and timepoints from 0 to 96 hours post ablation (hpa). Following photoreceptor (PR) ablation (red), activated Müller glia (MG, pink) form progenitors, which proliferate (purple) and differentiate into regenerated PRs (blue). (B-G”’) Micrographs showing the key timepoints and processes. PR (mCherry+, red) are ablated and progressively cleared from the retina. Proliferating progenitors (proliferative cell nuclear antigen, PCNA, pink) derived from Glutamine synthase (GS, green) expressing MG appear first at 24 hpa. These proliferative progenitors downregulate their mature MG markers as they form clonal chains of cells accompanying the regeneration progresses. DAPI labels nuclei (cyan). Scale bars: 50 μm.
(PDF)

S3 Fig Expression changes of Hes1/Her6 in the inner nuclear layer of regenerating zebrafish retina. (A-F”’) In adult retina, Hes1 (pink) is expressed in the inner half of the inner nuclear layer in the Müller glia (MG) and retinal neurons which express Glutamine synthase (GS). White dotted outlines represent examples of easily distinguishable MG. Following photoreceptor ablation (mCherry+) and as regeneration progresses, the expression of Hes1 is downregulated specifically in MG derived cells from 72 hpa. (H) Experimental design showing the timing of the metronidazole (MTZ) treatment and sample processing timeline. DAPI label nuclei (cyan). Scale bars: 50 μm.
(PDF)

S4 Fig Time course analysis of Müller glia derived regeneration in the larval zebrafish. (A) Schematic depicting the retinal layers in the larval zebrafish. (B-E”’) Ablation of photoreceptors (PRs, red) induced with metronidazole (MTZ) in Tg(*lws2*:*nfsb-mCherry*) in larval zebrafish follow a similar regenerative time-course to that of the adults. By 48 hours post ablation (hpa), Müller glia (MG, GFAP+, green) derived progenitors undergo proliferation, as indicated by PCNA (pink). DAPI label nuclei (cyan). Scale bars: 50 μm.
(PDF)

S5 Fig *her6* and *prox1a* MOs causes a downregulation of Hes1 and Prox1a proteins. (A-H”) In the adult retina, compared to standard control (SC) morpholino (MO) samples, downregulation of Prox1a is observed following *prox1a* MO electroporation and downregulation of Hes1 is observed following *her6* MO electroporation. DAPI labels nuclei (cyan). Glial fibrillary acidic protein (GFAP) labels the Müller glia (green). (I) Quantification demonstrates that the mosaic nature of the electroporation results in patches of efficient knockdown (see micrographs), but overall induces only a modest reduction in protein knockdown. Scale bars: 50 μm.
(PDF)

S6 Fig Her6 MO causes ablation of photoreceptors and prox1a MO induces an early burst of proliferation. (A-C”) In the adult retina, electroporation of *her6* morpholino (MO) causes efficient ablation of photoreceptors (loss of red mCherry labelled photoreceptors–red signal), compared to standard control (SC), suggesting that changes observed at later timepoints are due to differences in the regenerative process. (D-G’) Electroporation of *prox1a* MO causes an increase in mitotic cells marked by pH3 at 72 hpa, but not 120 hpa compared to SC MO in the INL and ONL, quantified in H. DAPI label nuclei (cyan). Scale bars: 50 μm.
(PDF)

S7 Fig Optimisation of liquid-liquid phase separate chemical disruption (1.5%). In the larval retina, metronidazole (MTZ) induced photoreceptor (mCherry+ red) ablation paradigm was used to optimise the length of exposure, concentration and duration of the drugs used to disrupt the liquid-liquid phase separants (LLPS) that contained Prospero homeobox1a Prox1a (pink) puncta. Müller glia are marked by GFAP (green). DAPI labels nuclei (cyan). (8A-L’) Expression of Prox1a in the outer nuclear layer (white line) showed modest reduction after exposure to 2, 10 or 30 min of 1.5% 1,6 HD when compared to 1.5% 2,5 HD control exposure or non-treated control samples.
(PDF)

S8 Fig Optimisation of liquid-liquid phase separate chemical disruption (5%). In the larval retina, metronidazole (MTZ) induced photoreceptor (mCherry+ red) ablation paradigm was used to optimise the length of exposure, concentration and duration of the drugs used to disrupt the liquid-liquid phase separants (LLPS) that contained Prospero homeobox1a Prox1a (pink) puncta. Müller glia are marked by GFAP (green). DAPI labels nuclei (cyan). (9A-L’) The expression of Prox1a in the ONL after exposure to 5% 1,6 HD at 2, 10 and 30 min is efficiently reduced, both when compared to 5% 2,5 HD control exposure or non-treated control samples. Thus 5%, 2 min treatment was chosen for the experiments.
(PDF)

S9 Fig Chemical disruption of liquid-liquid phase separates negatively affects the regeneration of cone photoreceptors. (A-H”’) Following photoreceptor (mCherry+) ablation in the larval retina, zpr-1 mature cone photoreceptor label (pink) was reduced following chemical exposure with 1,6-HD to disrupt LLPS, versus 2,5-HD as control at all timepoints shown from 24 to 96 hours post ablation (hpa). DAPI label nuclei (cyan). Scale bars: 50 μm.
(PDF)
