# Supplementary material for: Her6 and Prox1a are novel regulators of photoreceptor regeneration in the zebrafish retina
Source: PLoS Genet. 2023 Nov 6;19(11):e1011010. doi: 10.1371/journal.pgen.1011010 (PMC10653607; doi:10.1371/journal.pgen.1011010)
Supplement: S1 Table — The name of the gene, location of expression, types of gene manipulation (overexpression (OE) / RNAi) and result of screen under both GMRH108Gal4 and hs FLIP actin promoters are shown. Grey indicates the candidate genes that were screened. Green indicates that Miranda positive ectopic stem cells were identified. The misexpression of deadpan and prospero induced Miranda positive cells under both promoters. The knockdown of SoxNeuro and elav induced Miranda positive cells under the hsFLIP actin promoter. (PDF) [file pgen.1011010.s001.pdf]

Gene	Expression	OE/ RNAi	*GMRH108Gal4>* phenotype	Heat shock flip out clones phenotype
asense	Neuroblasts	OE	n	n
		RNAi	n	n
chinmo	Neurons	RNAi	n	n
deadpan	Neuroblasts	OE	y	y
dichaete	Neuroblasts	RNAi	n	n
elav	Neurons	RNAi	n	y
erect wing	Neurons	RNAi	n	n
grainy head	Neuroblasts	RNAi	n	n
inscuteable	Neuroblasts	N/A	n/a	n/a
klumpfuss	Neuroblasts	N/A	n/a	n/a
Lim3	Neurons	RNAi	n	n
minibrain	Neurons	RNAi	n	n
miranda	Neuroblasts	N/A	n/a	n/a
ocelliless	Neurons	RNAi	n/a	n/a
prospero	Neurons	RNAi	y	y
roundabout3	Neurons	N/A	n/a	n/a
SoxNeuro	Neurons	OE	n	n/a
		RNAi	n	y
worniu	Neuroblasts	N/A	n/a	n/a
zelda	Neuroblasts	RNAi	n	n
