# Supplementary figures and images for: Her6 and Prox1a are novel regulators of photoreceptor regeneration in the zebrafish retina
Source: PLoS Genet. 2023 Nov 6;19(11):e1011010. doi: 10.1371/journal.pgen.1011010 (PMC10653607; doi:10.1371/journal.pgen.1011010)

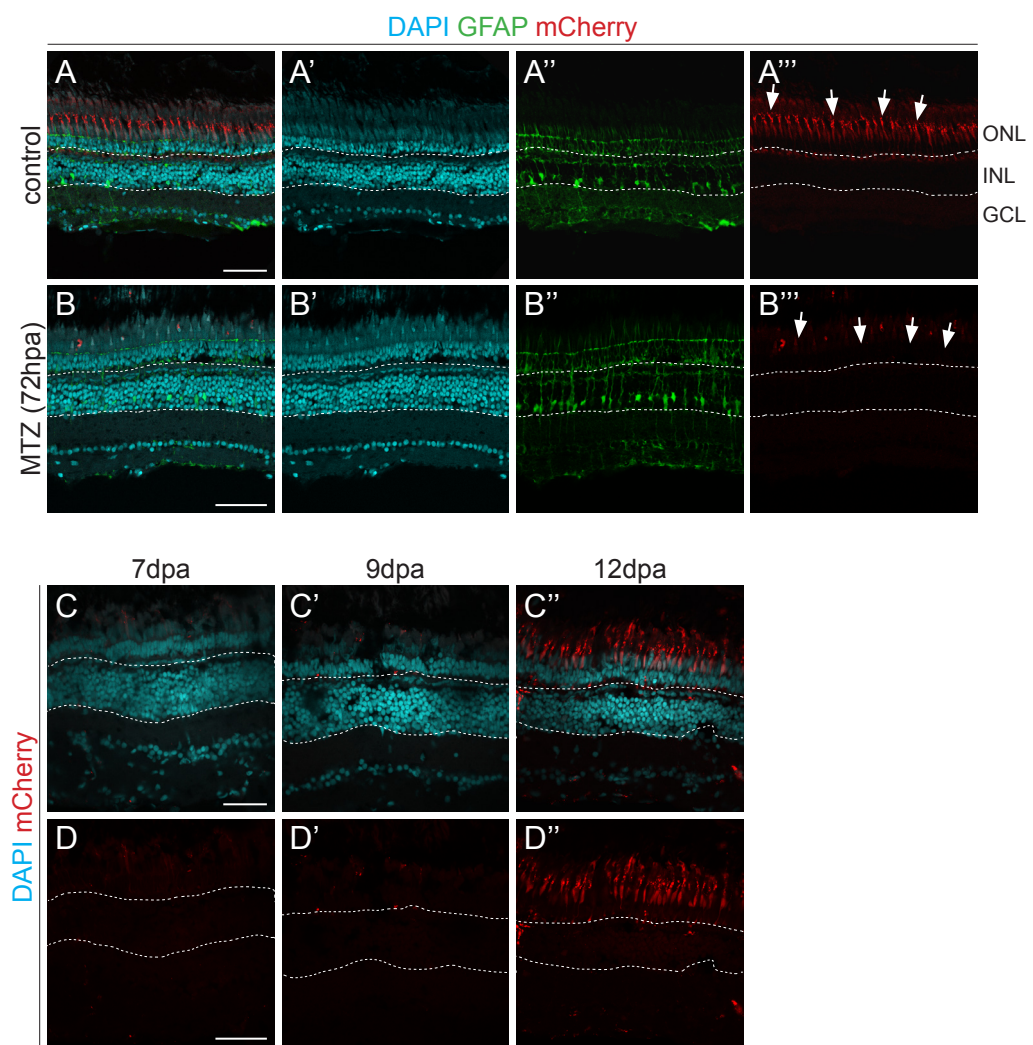

Supplement: S1 Fig — (A-B”’) Metronidazole (MTZ) induced ablation of mCherry labelled photoreceptors in Tg(lws2:nfsb-mCherry, gfap:eGFP) adult zebrafish efficiently ablated the targeted cells as shown at 72 hours post ablation (hpa). (C-D”) Expression of the lws2 promoter driven mCherry in regenerated cells was re-established by 12 days post ablation (dpa). DAPI labels nuclei (cyan) and Glial fibrillary acidic protein (GFAP, green) marks Müller glia. Scale bars: 50 μm. (PDF) [file pgen.1011010.s002.pdf]

A

Hours post ablation (hpa)

0                      16                      24                      48                      72                      96

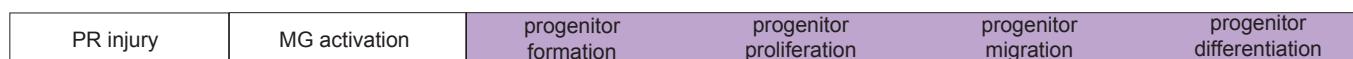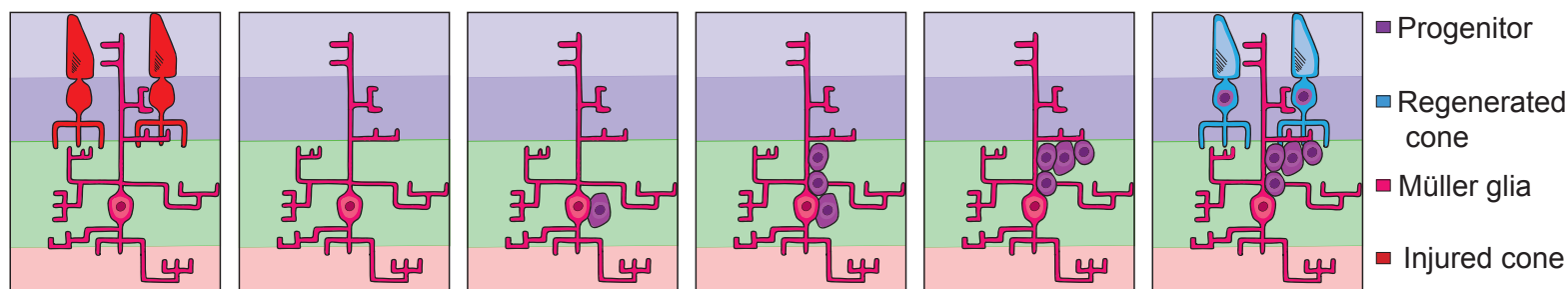

control

16hpa

24hpa

48hpa

72hpa

96hpa

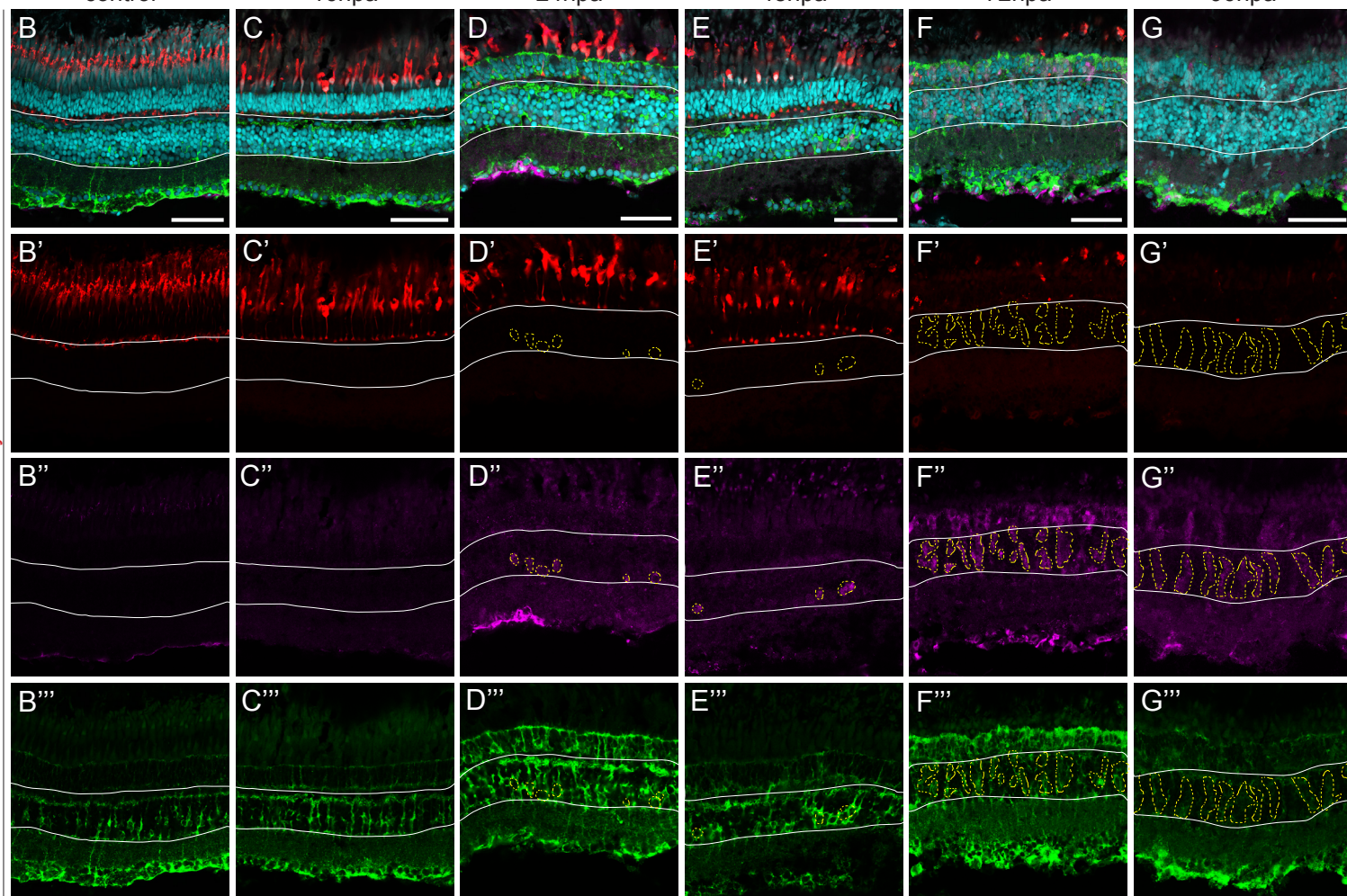

Supplement: S2 Fig — (A) Summary of adult retinal regeneration stages and timepoints from 0 to 96 hours post ablation (hpa). Following photoreceptor (PR) ablation (red), activated Müller glia (MG, pink) form progenitors, which proliferate (purple) and differentiate into regenerated PRs (blue). (B-G”’) Micrographs showing the key timepoints and processes. PR (mCherry+, red) are ablated and progressively cleared from the retina. Proliferating progenitors (proliferative cell nuclear antigen, PCNA, pink) derived from Glutamine synthase (GS, green) expressing MG appear first at 24 hpa. These proliferative progenitors downregulate their mature MG markers as they form clonal chains of cells accompanying the regeneration progresses. DAPI labels nuclei (cyan). Scale bars: 50 μm. (PDF) [file pgen.1011010.s003.pdf]

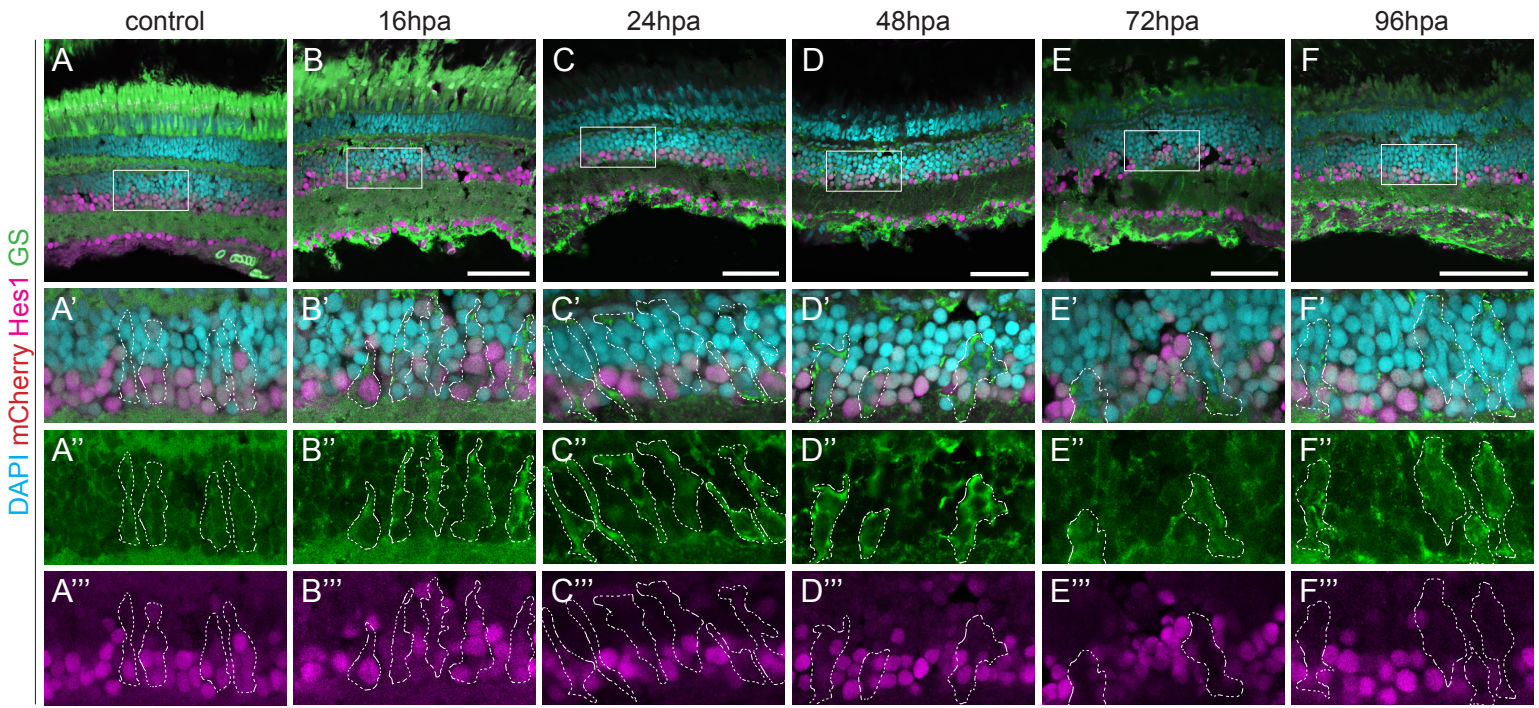

H

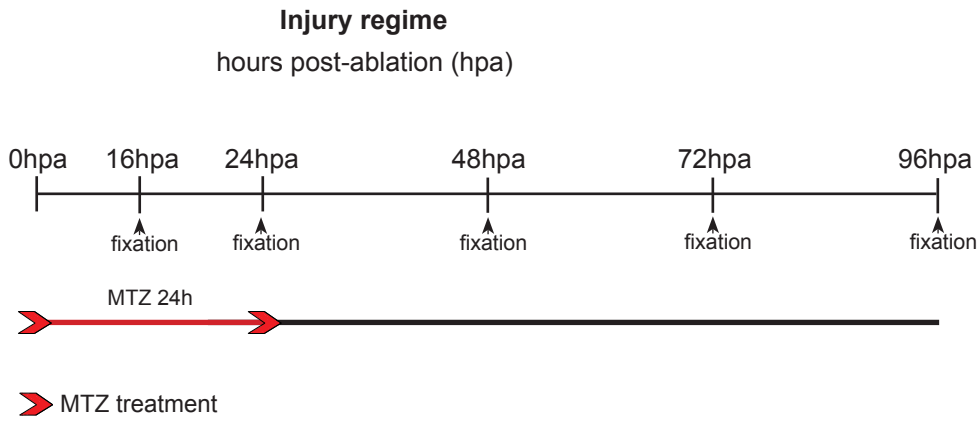

Supplement: S3 Fig — (A-F”’) In adult retina, Hes1 (pink) is expressed in the inner half of the inner nuclear layer in the Müller glia (MG) and retinal neurons which express Glutamine synthase (GS). White dotted outlines represent examples of easily distinguishable MG. Following photoreceptor ablation (mCherry+) and as regeneration progresses, the expression of Hes1 is downregulated specifically in MG derived cells from 72 hpa. (H) Experimental design showing the timing of the metronidazole (MTZ) treatment and sample processing timeline. DAPI label nuclei (cyan). Scale bars: 50 μm. (PDF) [file pgen.1011010.s004.pdf]

A

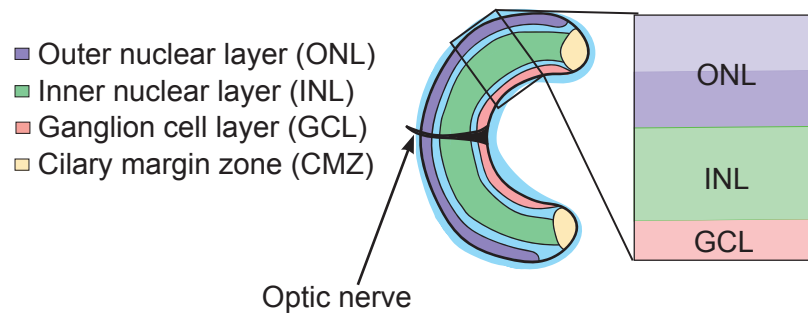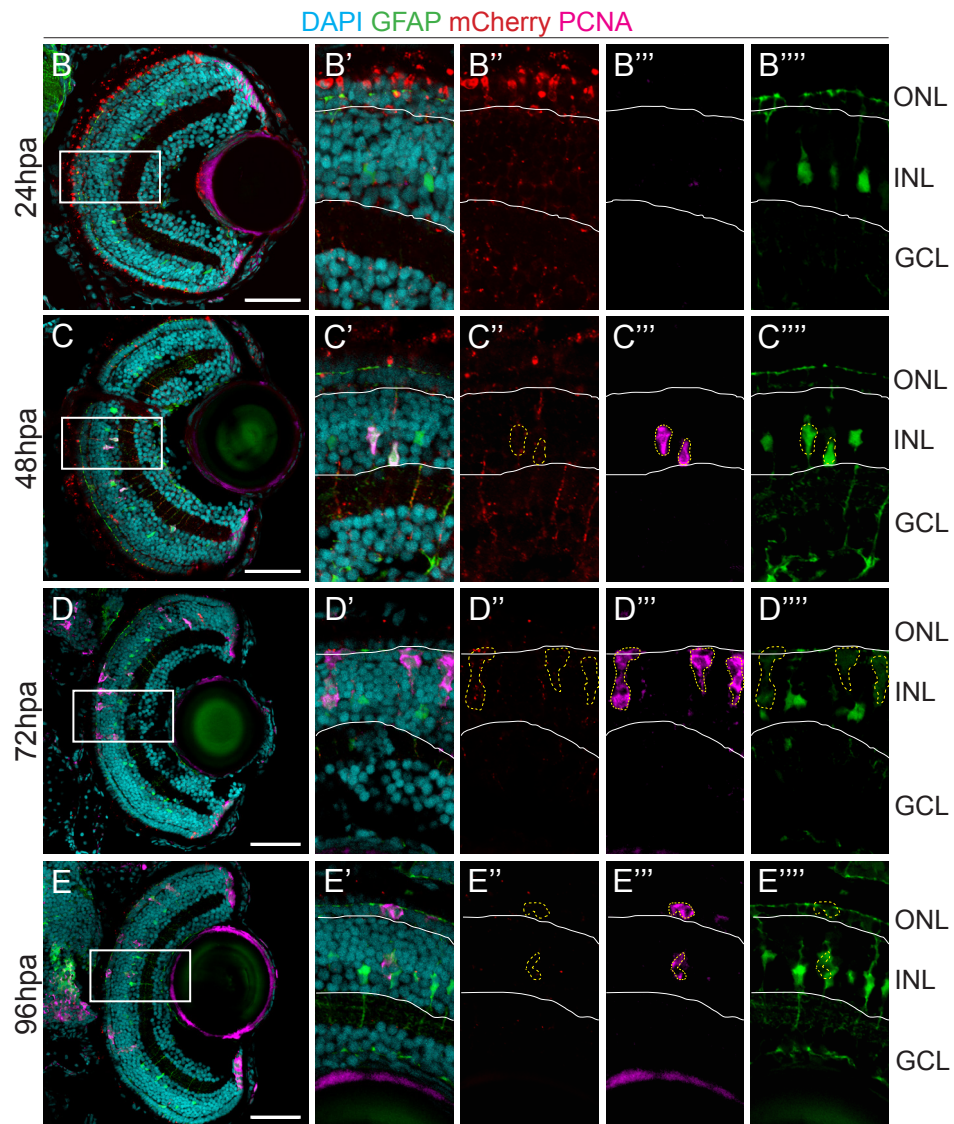

Supplement: S4 Fig — (A) Schematic depicting the retinal layers in the larval zebrafish. (B-E”’) Ablation of photoreceptors (PRs, red) induced with metronidazole (MTZ) in Tg(lws2:nfsb-mCherry) in larval zebrafish follow a similar regenerative time-course to that of the adults. By 48 hours post ablation (hpa), Müller glia (MG, GFAP+, green) derived progenitors undergo proliferation, as indicated by PCNA (pink). DAPI label nuclei (cyan). Scale bars: 50 μm. (PDF) [file pgen.1011010.s005.pdf]

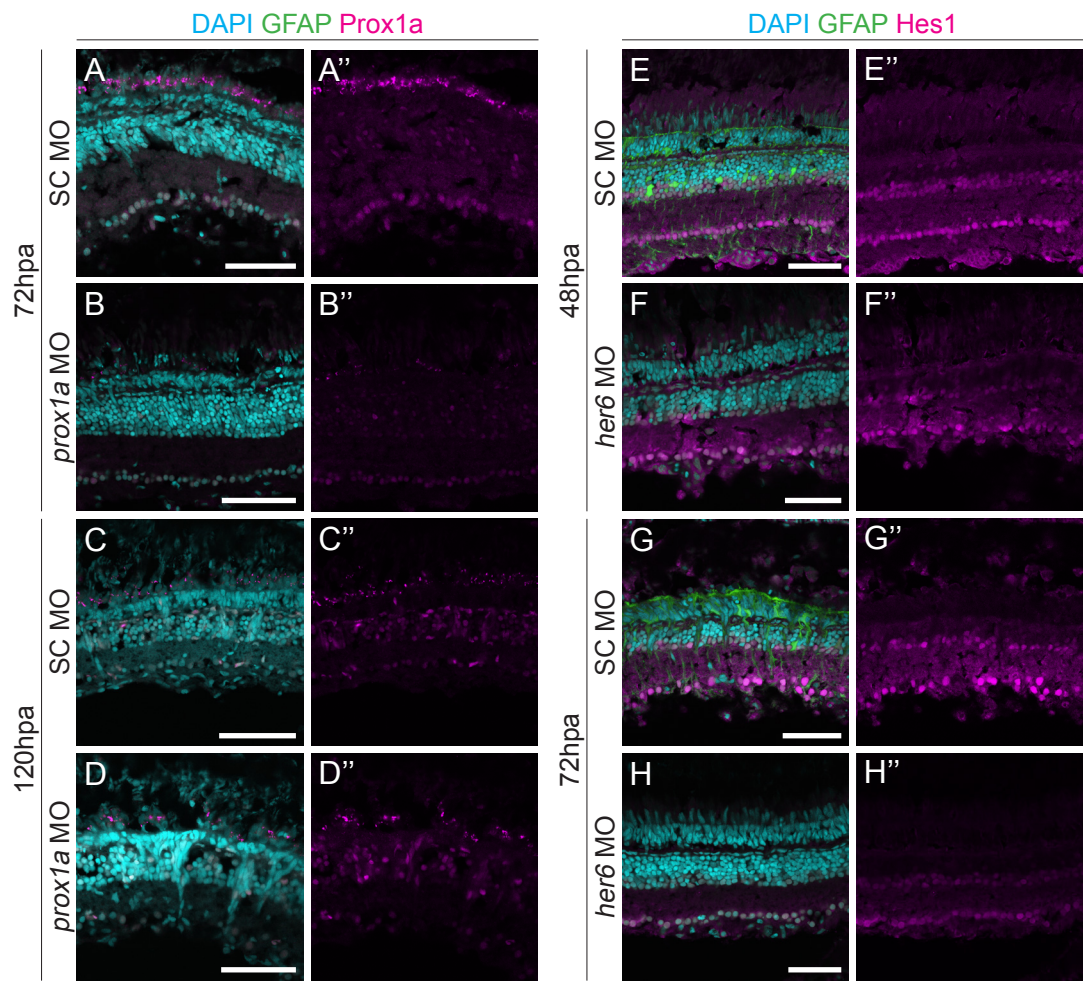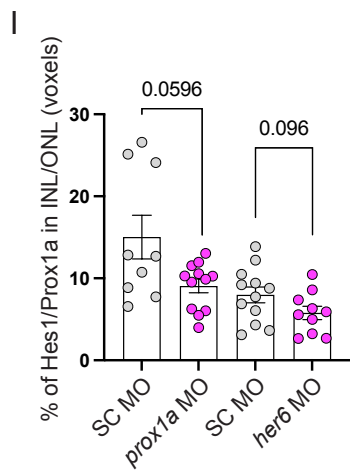

Supplement: S5 Fig — (A-H”) In the adult retina, compared to standard control (SC) morpholino (MO) samples, downregulation of Prox1a is observed following prox1a MO electroporation and downregulation of Hes1 is observed following her6 MO electroporation. DAPI labels nuclei (cyan). Glial fibrillary acidic protein (GFAP) labels the Müller glia (green). (I) Quantification demonstrates that the mosaic nature of the electroporation results in patches of efficient knockdown (see micrographs), but overall induces only a modest reduction in protein knockdown. Scale bars: 50 μm. (PDF) [file pgen.1011010.s006.pdf]

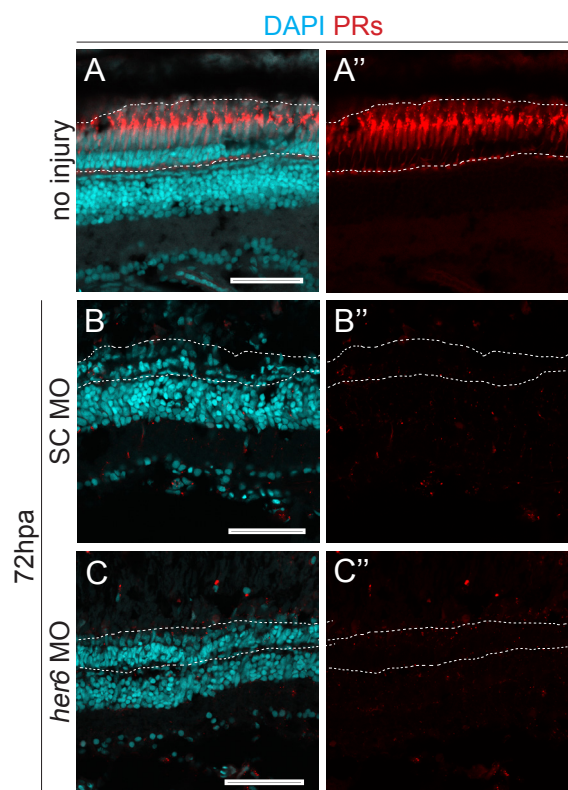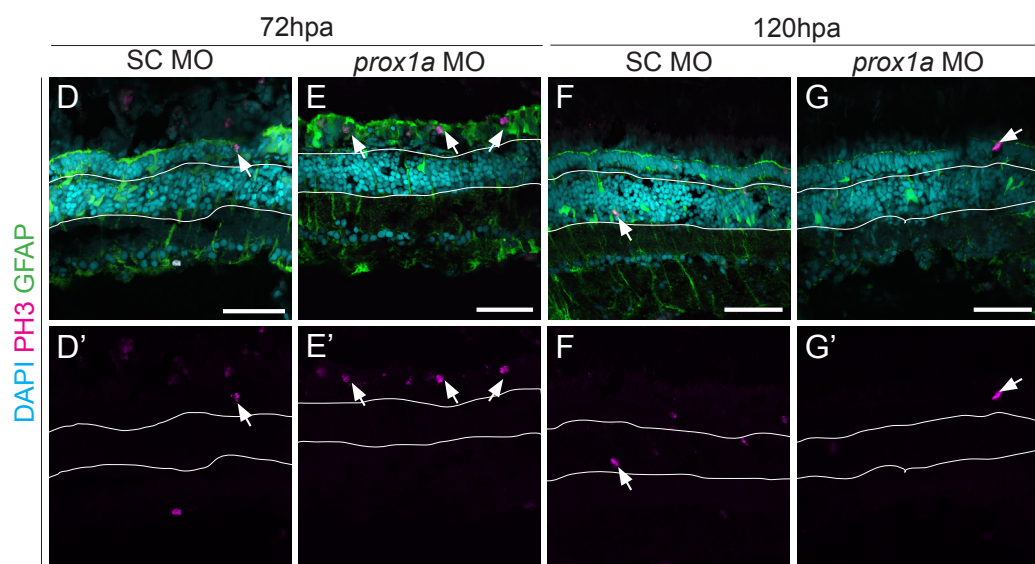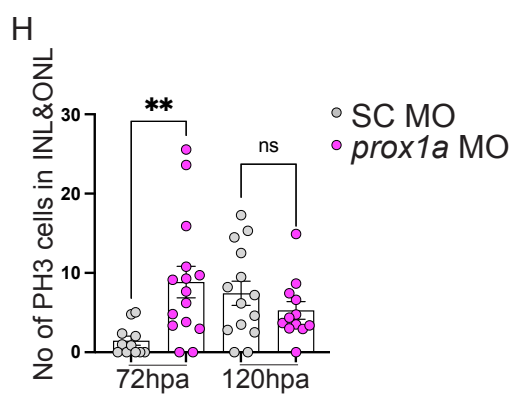

Supplement: S6 Fig — (A-C”) In the adult retina, electroporation of her6 morpholino (MO) causes efficient ablation of photoreceptors (loss of red mCherry labelled photoreceptors–red signal), compared to standard control (SC), suggesting that changes observed at later timepoints are due to differences in the regenerative process. (D-G’) Electroporation of prox1a MO causes an increase in mitotic cells marked by pH3 at 72 hpa, but not 120 hpa compared to SC MO in the INL and ONL, quantified in H. DAPI label nuclei (cyan). Scale bars: 50 μm. (PDF) [file pgen.1011010.s007.pdf]

DAPI GFAP mCherry Prox1a

1.5% 1,6-HD

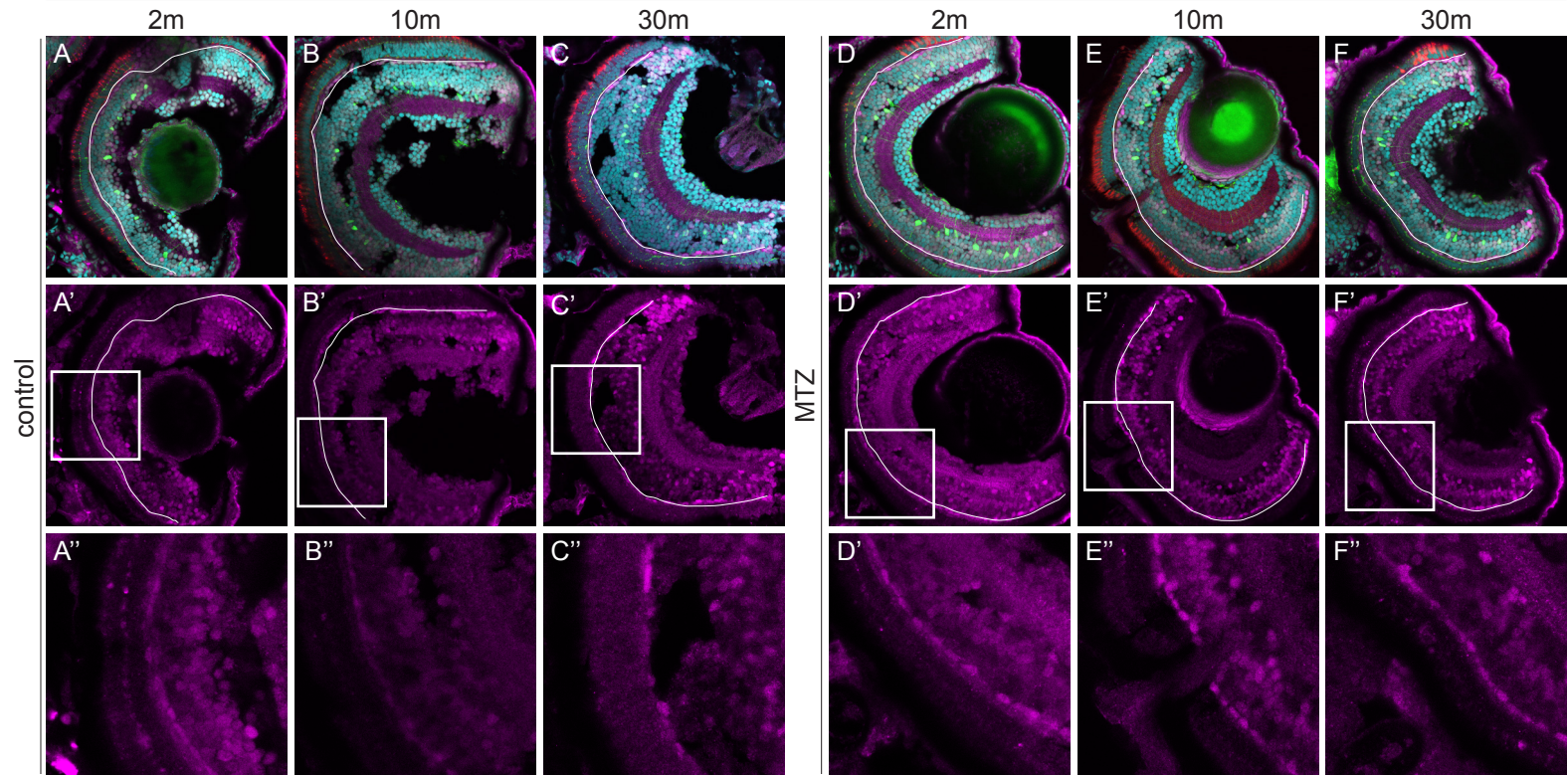

1.5% 2,5-HD

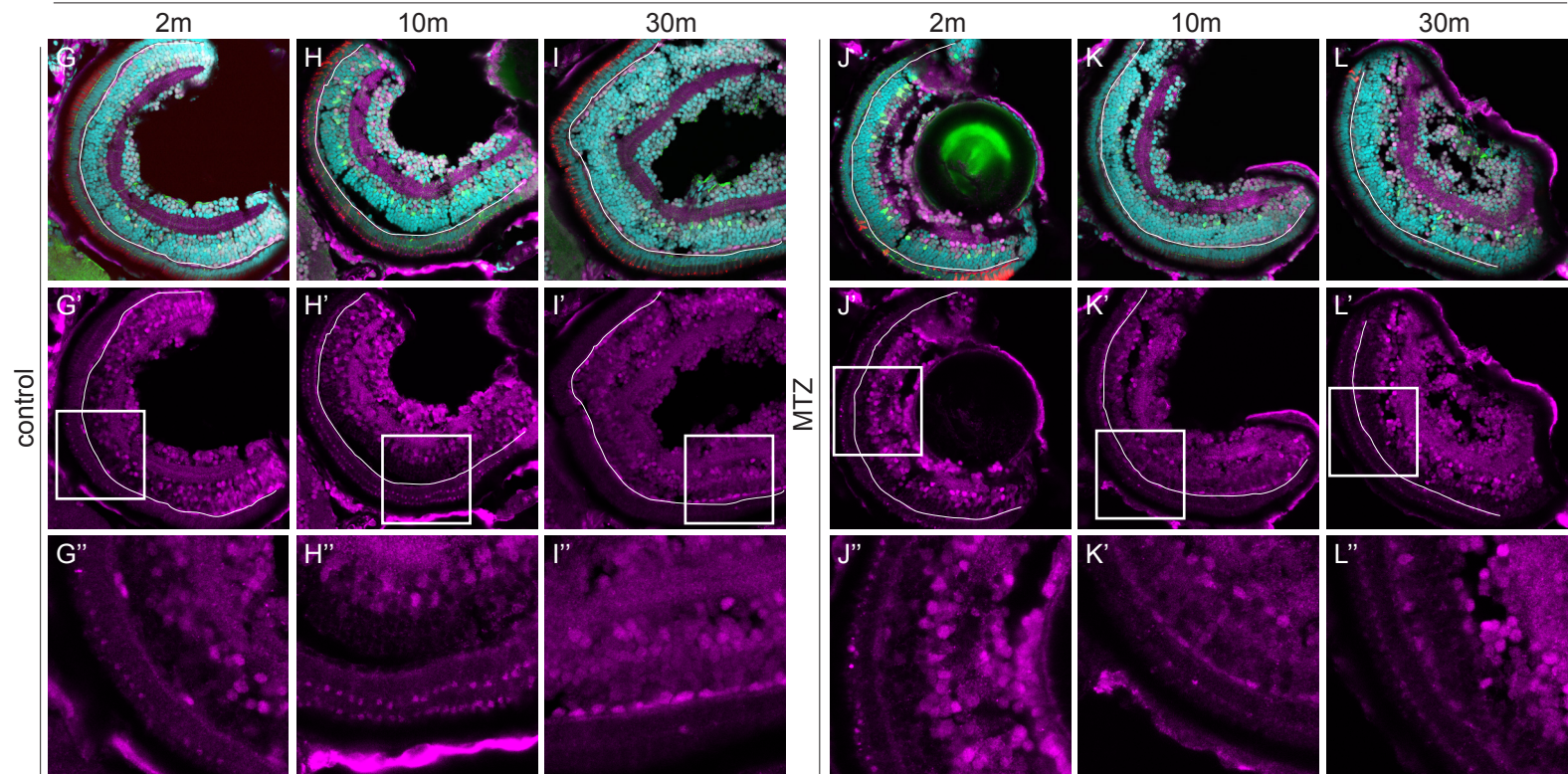

Supplement: S7 Fig — In the larval retina, metronidazole (MTZ) induced photoreceptor (mCherry+ red) ablation paradigm was used to optimise the length of exposure, concentration and duration of the drugs used to disrupt the liquid-liquid phase separants (LLPS) that contained Prospero homeobox1a Prox1a (pink) puncta. Müller glia are marked by GFAP (green). DAPI labels nuclei (cyan). (8A-L’) Expression of Prox1a in the outer nuclear layer (white line) showed modest reduction after exposure to 2, 10 or 30 min of 1.5% 1,6 HD when compared to 1.5% 2,5 HD control exposure or non-treated control samples. (PDF) [file pgen.1011010.s008.pdf]

DAPI GFAP mCherry Prox1a

5% 1,6-HD

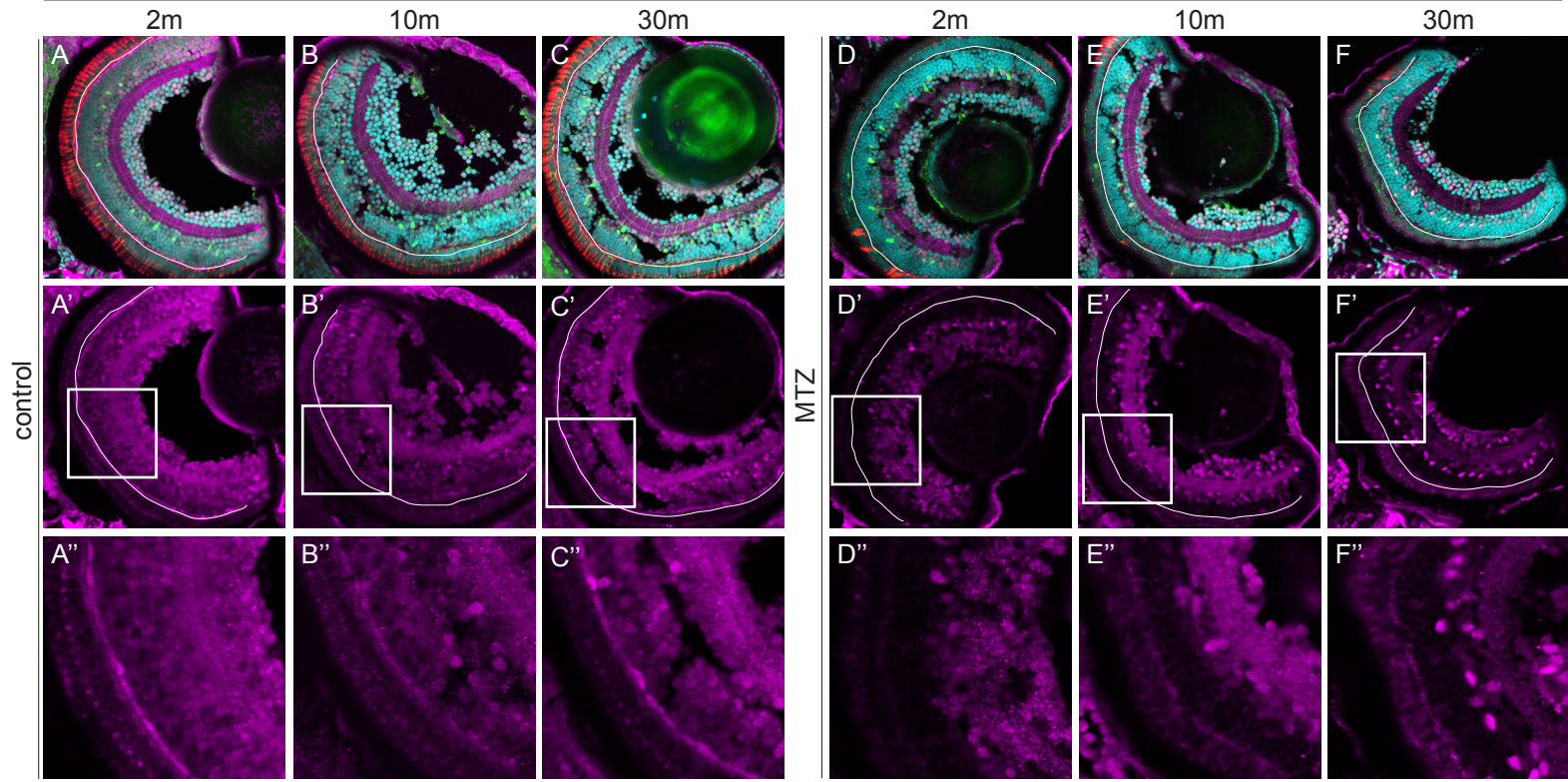

5% 2,5-HD

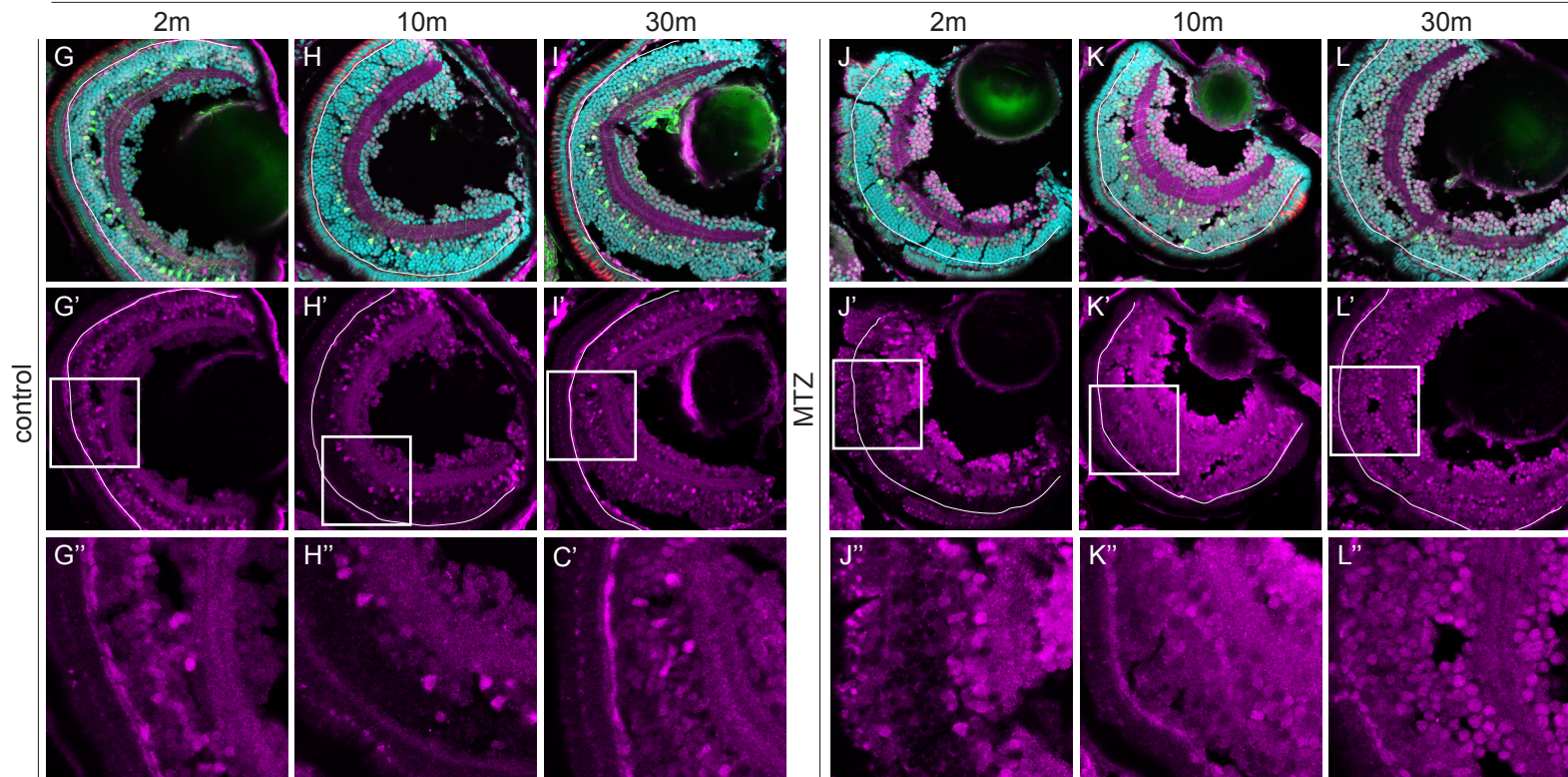

Supplement: S8 Fig — In the larval retina, metronidazole (MTZ) induced photoreceptor (mCherry+ red) ablation paradigm was used to optimise the length of exposure, concentration and duration of the drugs used to disrupt the liquid-liquid phase separants (LLPS) that contained Prospero homeobox1a Prox1a (pink) puncta. Müller glia are marked by GFAP (green). DAPI labels nuclei (cyan). (9A-L’) The expression of Prox1a in the ONL after exposure to 5% 1,6 HD at 2, 10 and 30 min is efficiently reduced, both when compared to 5% 2,5 HD control exposure or non-treated control samples. Thus 5%, 2 min treatment was chosen for the experiments. (PDF) [file pgen.1011010.s009.pdf]

DAPI GFAP mCherry zpr1

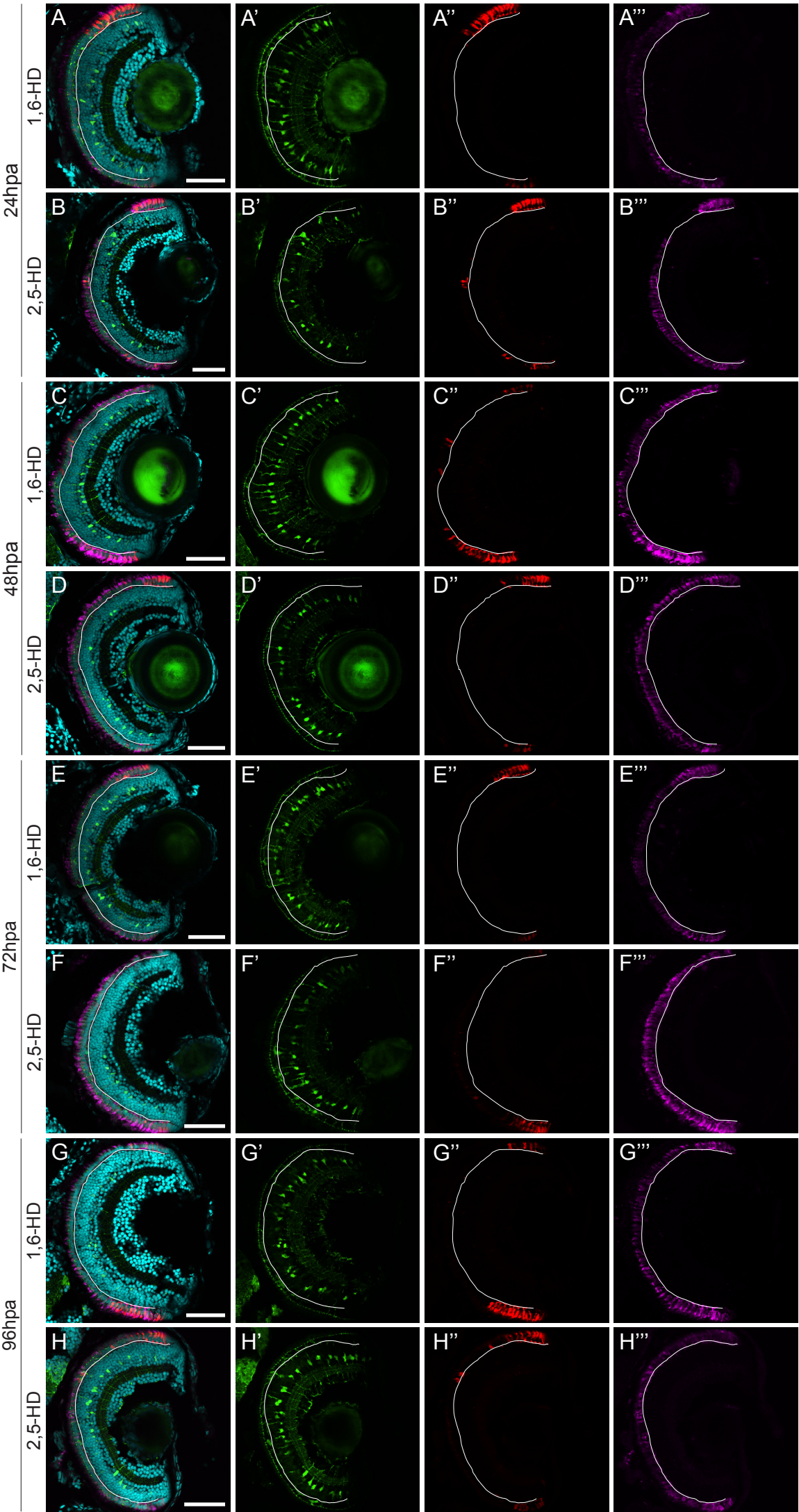

Supplement: S9 Fig — (A-H”’) Following photoreceptor (mCherry+) ablation in the larval retina, zpr-1 mature cone photoreceptor label (pink) was reduced following chemical exposure with 1,6-HD to disrupt LLPS, versus 2,5-HD as control at all timepoints shown from 24 to 96 hours post ablation (hpa). DAPI label nuclei (cyan). Scale bars: 50 μm. (PDF) [file pgen.1011010.s010.pdf]
